# Overcoming Resistance: Targeting Survivin-Driven Apoptotic Resistance Restores Irinotecan Sensitivity in *TP53*-Mutant Colorectal Cancer

**DOI:** 10.3390/cancers18162687

**Published:** 2026-08-19

**Authors:** Daciana Catalina Dumut, Yong Zhong Xu, Daniela Verelli, Viswanath Das, Marian Hajduch, Juan Bautista De Sanctis, Danuta Radzioch

**Affiliations:** 1 Department of Experimental Medicine, Faculty of Medicine, McGill University, Montreal, QC H3A 1Y2, Canada; daciana.dumut@mail.mcgill.ca (D.C.D.); yong.xu@mail.mcgill.ca (Y.Z.X.); 2 Infectious Diseases in Global Health Program, The Research Institute of the McGill University Health Centre, Montreal, QC H3A 1A1, Canada; daniela.verelli@mail.mcgill.ca; 3 Department of Microbiology and Immunology, Faculty of Medicine, McGill University, Montreal, QC H3A 0G4, Canada; 4 Institute of Molecular and Translational Medicine, Faculty of Medicine and Dentistry, Palacky University, 77900 Olomouc, Czech Republic; viswanath.das@upol.cz (V.D.); marian.hajduch@upol.cz (M.H.); juanbautista.desanctis@upol.cz (J.B.D.S.); 5 Czech Advanced Technology and Research Institute, Palacky University Olomouc, Krizkovskeho 511/8, 77900 Olomouc, Czech Republic

**Keywords:** colorectal cancer, *TP53*, Survivin inhibitor, disulfiram, bis(diethyldithiocarbamato)copper, CuET, YM-155, SN-38, irinotecan, apoptosis, chemoresistance

## Abstract

**Background/Objectives:** Metastatic colorectal cancer (mCRC) remains difficult to treat, largely due to chemotherapy resistance. Mutations in *TP53* impair apoptosis and are associated with poor response to irinotecan. This study aimed to determine whether targeting Survivin, a key inhibitor of apoptosis, and using the disulfiram-derived compound CuET could restore apoptotic signaling and improve irinotecan efficacy. **Methods:** Human CRC cell lines with varying *TP53* status and murine tumor models were treated with irinotecan (or its active metabolite SN-38), the Survivin inhibitor YM-155, and CuET, alone or in combination. Cell viability, clonogenic survival, and apoptotic signaling were assessed using cytotoxicity assays, flow cytometry, confocal microscopy, and Western blotting. *In vivo* efficacy was evaluated in xenograft and syngeneic mouse models through tumor growth measurements and histological analyses. **Results:** Inhibition of Survivin with YM-155 enhanced irinotecan-induced cytotoxicity and promoted caspase-dependent apoptosis in CRC cells. Irinotecan treatment induced Survivin expression, suggesting an adaptive resistance mechanism that was reversed by YM-155. CuET demonstrated potent cytotoxic activity independent of *TP53* status and partially suppressed Survivin expression. Importantly, CuET restored sensitivity to irinotecan in *TP53*-deficient models and significantly enhanced antitumor efficacy *in vivo*, leading to reduced tumor growth, decreased proliferation, and increased apoptosis. **Conclusions:** These findings identify Survivin-mediated apoptotic resistance as a key determinant of irinotecan response in CRC. Targeting this pathway, either directly through Survivin inhibition or through CuET-induced stress responses, restores apoptotic sensitivity and enhances chemotherapy efficacy. This study supports the development of combination strategies incorporating CuET to overcome resistance in *TP53*-mutant CRC.

## Simple Summary

Colorectal cancer is one of the leading causes of cancer-related deaths, especially when it spreads to other parts of the body. A major challenge in treatment is that many tumors become resistant to chemotherapy, particularly in patients whose tumors carry mutations in *TP53*. These mutations make cancer cells less likely to undergo programmed cell death, allowing them to survive treatment. One protein, Survivin, plays a key role in this resistance by helping cancer cells avoid death. In this study, we explored whether targeting Survivin and using CuET, a compound derived from an existing drug, could restore the ability of cancer cells to respond to treatment. Our findings suggest that combining this compound with standard chemotherapy may help overcome resistance and improve treatment outcomes, offering a potential new strategy for patients with difficult-to-treat colorectal cancer.

## 1. Introduction

Colorectal cancer (CRC) is the second leading cause of cancer-related mortality worldwide, and therapy resistance remains a major obstacle in the management of advanced, metastatic disease [1]. CRC arises through a process known as “multistep tumorigenesis”, characterized by the sequential accumulation of genomic alterations in tumor suppressor genes and in oncogenes.

Early events in CRC development include the loss of *APC*, followed by activating mutations in *KRAS* or *BRAF*. Additionally, chromosomal instability arises from defects in the DNA-repair and chromosome-segregation mechanisms. Defects in DNA-replication proofreading further contribute to CRC heterogeneity [2]. The acquisition of *TP53* mutations typically occurs later in the process and promotes malignant progression and metastatic dissemination [3]. Standard therapeutic regimens for metastatic CRC (mCRC), such as FOLFOX and FOLFIRI, incorporate the DNA-damaging agents 5-fluorouracil, oxaliplatin, and the topoisomerase I inhibitor irinotecan (CPT-11). Despite these available treatments, the 5-year survival rate for mCRC remains below 15%, highlighting the urgent need to overcome therapeutic resistance that limits treatment efficacy [4].

Chemoresistance in CRC arises from diverse molecular alterations, particularly those that disrupt apoptotic signaling. Among these, *TP53* mutations—present in approximately 50–60% of CRCs—are strongly associated with reduced sensitivity to irinotecan and other DNA-damaging chemotherapies [5]. Multiple studies have shown that loss or mutation of p53 impairs canonical DNA-damage-induced cell-cycle arrest and apoptotic signaling, thereby enabling tumor cell survival following genotoxic injury and potentially reducing the efficacy of irinotecan-based treatment [6,7,8,9].

A major driver of chemoresistance in *TP53*-mutated CRC is the disruption of the p53–Survivin regulatory axis. Specifically, wild-type p53 represses the transcription of *BIRC5* (Survivin), a member of the inhibitor-of-apoptosis (IAP) family that suppresses caspase activation and supports mitotic progression despite DNA-damage. When p53 function is lost or altered, the repression of *BIRC5* is relieved, resulting in aberrantly high Survivin expression and the reinforcement of anti-apoptotic signaling including NF-κB and BCL-2, which contribute to resistance across diverse CRC subtypes [10,11].

Although Survivin is normally downregulated in terminally differentiated cells, its expression is elevated in many malignancies and in cells responding to radiation-induced DNA damage [10,12]. In CRC, Survivin serves as an important prognostic marker: higher expression levels are associated with tumor progression and reduced patient survival [13,14,15]. Consistent with these clinical observations, the role of Survivin as a mediator of chemoresistance is well established [16,17,18,19,20,21,22,23]. Accordingly, therapeutic strategies that target Survivin or engage p53-independent apoptotic pathways offer a promising approach to resensitize CRC cells to irinotecan.

Multiple groups have explored radio- and chemosensitization strategies by targeting Survivin using antisense oligonucleotides, small interfering (si)RNAs, CRISPR-Cas9-mediated knockouts, and small-molecule inhibitors [23,24,25,26,27,28,29]. Although these approaches have shown promising preclinical efficacy, Survivin-directed therapies have yet to demonstrate durable clinical benefit, limiting their progression into late-phase clinical development. Notably, sepantronium bromide (YM-155), a small-molecule Survivin inhibitor, demonstrated acceptable safety in phase I studies but produced only modest improvements in progression-free survival in phase II trials across multiple solid tumors [30,31,32,33]. These limited clinical responses suggest that Survivin inhibition alone may be insufficient for durable tumor control because tumors can maintain survival through redundant anti-apoptotic and stress-response pathways. This provides a rationale for combining Survivin-targeted agents with cytotoxic chemotherapy or complementary p53-independent therapies, as suppression of adaptive Survivin signaling may enhance the effects of treatment-induced DNA damage.

The disulfiram metabolite bis(diethyldithiocarbamato)copper (CuET) has demonstrated potent anticancer activity across multiple preclinical models. CuET targets the p97–NPL4 complex of the ubiquitin–proteasome system, leading to proteotoxic stress through accumulation of misfolded proteins [34]. Importantly, this mechanism is p53-independent [35,36]. Additionally, disulfiram/copper has been shown to suppress NF-κB signaling, reducing anti-apoptotic gene expression such as *BCL2*, *XIAP*, and *BIRC5* [37,38]. By blocking NF-κB nuclear translocation and transcriptional activation, CuET may downregulate these survival mediators, thereby reversing apoptotic resistance in p53-mutant CRC. Consequently, combining CuET with irinotecan offers a rational dual-targeting strategy in *TP53*-mutated CRC, coupling irinotecan-induced DNA damage with CuET-driven proteotoxic stress to bypass defective p53 signaling and overcome irinotecan resistance. This mechanism is depicted in the Graphical Abstract below.

In this study, we investigate whether inhibiting Survivin with YM-155 can enhance the efficacy of irinotecan (SN-38 *in vitro* and CPT-11 *in vivo*) in CRC. We further examine the therapeutic potential and mechanistic basis of combining CuET and irinotecan in *TP53*-mutant CRC, using it as a second, mechanistically distinct strategy to address the same resistance phenotype in a setting where p53-dependent apoptosis is impaired. We test the hypothesis that irinotecan sensitivity can be restored in *TP53*-mutant CRC by inhibiting Survivin and reactivating caspase-dependent apoptosis with CuET treatment. In addition to demonstrating synergy and enhanced CRC cell elimination when YM-155 is combined with SN-38, our findings show for the first time that CuET not only overcomes irinotecan resistance but also amplifies cancer cell apoptosis, supporting its translational promise as a novel therapeutic strategy for refractory, *TP53*-mutant CRC.

## 2. Materials and Methods

### 2.1. Drugs

YM-155 (sepantronium bromide; #6491), the CPT-11 metabolite SN-38 (#2684), and CPT-11 (irinotecan; #2688) were purchased from Tocris Bioscience (Bristol, UK). CuET (copper (II) diethyldithiocarbamate; #D0487) was purchased from Tokyo Chemical Industry (Tokyo, Japan). All drugs used in the *in vitro* studies were reconstituted in DMSO (Sigma, St. Louis, MO, USA). Vehicle concentrations were normalized across treatment groups and did not exceed 0.1% DMSO.

### 2.2. Cell Culture

The human CRC cell lines HCT116 Parental (*KRAS^G13D^/KRAS^WT^*, ATCC CCL-247 via Horizon #HD PAR-007), HCT116 WT (*KRAS^WT^/KRAS^KO^*, #HD-104-008), HCT116 *KRAS^G13D^* (*KRAS^G13D^/KRAS^KO^*, #HD 104-011), and HCT116 *TP53^−/−^* (HD 104-001) were maintained in McCoy’s 5A medium. The human CRC cell line HT-29 *TP53^R273H^* (ATCC HTB-38) was maintained in McCoy’s 5A medium. The human DLD-1 *TP53^S241F/−^* cells/spheroids (#HD 105-014) were maintained in RPMI-1640 medium. The murine CRC cell line MC-38, which harbors two heterozygous *Trp53* missense mutations (G242V and S258I) were kindly provided by Dr. Pnina Brodt (McGill University, Montréal, QC, Canada), who obtained it from Dr. Shoshana Yakar (New York University, New York, NY, USA). MC-38 cells were maintained in Dulbecco’s Modified Eagle Medium (DMEM). All cell lines were supplemented with 10% (*v*/*v*) fetal bovine serum (FBS), 1% (*v*/*v*) penicillin/streptomycin, and cultured in a humidified atmosphere of 5% CO_2_ at 37 °C. All cell culture reagents were purchased from Wisent (Quebec, QC, Canada).

### 2.3. Cell Proliferation and Cytotoxicity Assay

Cells were detached with 0.05% trypsin-EDTA and seeded at 5 × 10^3^ cells per well in 96-well plates. After 24 h, the cells were treated with serial dilutions of SN-38 and YM-155, alone and in combination, reconstituted in DMSO (Sigma). The final DMSO concentration was normalized across all wells and did not exceed 0.1%. Cells were incubated at 37 °C, 5% CO_2_ for 72 h. Cell viability was assessed using the XTT assay as per the manufacturer’s instructions (Invitrogen™, Carlsbad, CA, USA, CyQUANT™ XTT Cell Viability Assay, #X12223). Combination indices were calculated using the Chou–Talalay method, with CI < 1 indicating synergy, CI = 1 an additive interaction, and CI > 1 antagonism.

For the sulforhodamine B (SRB) cytotoxicity assay, cells were seeded as described for XTT and treated with serial dilutions of SN-38 and CuET. After 72 h, cells were fixed with 50% (*w*/*v*) trichloroacetic acid (Sigma) and stained for 30 min with 0.4% (*w*/*v*) sulforhodamine B (SRB; Alfa Aesar, Heysham, UK) dissolved in 1% (*w*/*v*) acetic acid (Sigma). Unbound dye was removed with four washes of 1% acetic acid solution, and protein-bound dye was solubilized using 10 mM unbuffered Tris base (Thermo Fisher Scientific, Waltham, MA, USA). Absorbance was measured at 492 nm. IC_50_ values were calculated by determining the percentage of cells killed relative to the DMSO control using the formula: % cells killed = 100 − (mean OD sample)/(mean OD control) × 100. Dose–response curves relating compound concentration to percent growth inhibition were plotted.

### 2.4. Clonogenic Assay

The cells were seeded at 300 cells per well in 6-well plates and treated with SN-38, YM-155, and CuET in DMSO every second day for seven days. Cultures were maintained at 37 °C with 5% CO_2_. On day 7, cells were washed and stained with 0.5% (*w*/*v*) crystal violet (Sigma-Aldrich, St. Louis, MO, USA) prepared in 70% ethanol. The total number of colonies was then visually quantified.

### 2.5. Tumor Spheroid Generation, Treatment, and Imaging

Multicellular tumor spheroids were generated using a modified liquid-overlay method in agarose-coated 384-well plates [39,40]. Cells were seeded into CellCarrier clear-bottom 384-well plates coated with 0.75% low-melting-point agarose in complete growth medium and allowed to self-aggregate under optimized conditions. The culture medium was replaced every three days. HCT116 Parental and *TP53^−/−^* spheroids were treated with SN-38, YM-155, or the indicated SN-38 plus YM-155 combinations, whereas DLD-1 spheroids were treated with CuET at the indicated concentrations and time points. Bright-field z-stack images were acquired using a fully automated CellVoyager CV7000 High-Content Imaging System (Yokogawa Electric Corporation, Tokyo, Japan) equipped with a 4× air objective. Images were acquired at 10–20 µm z-stack intervals using 4 × 4 binning and an exposure time of 11 ms. Spheroid images were analyzed using an in-house MATLAB R2026a-based semi-automated routine. The best-focused image was selected from each z-stack using an image-gradient criterion, after which the well boundary was cropped, and the spheroid was localized using a predefined circular filter. Spheroids were segmented from the background by thresholding, and cross-sectional area, major and minor axes, perimeter, and solidity were extracted. For quantification of spheroid growth, the cross-sectional area of each treated spheroid was normalized to the mean area of the corresponding untreated or vehicle-treated spheroids at the same time point. Data were expressed as a percentage of the untreated control, which was set to 100%. Segmentation of partially disintegrated drug-treated spheroids was manually corrected when required.

### 2.6. Western Blotting

Cells were seeded in 60-mm dishes at 8 × 10^5^ cells per dish. On the following day, the cells were treated with SN-38, YM-155, and CuET in DMSO for 24, 48, and 72 h. Cells were then lysed in radioimmunoprecipitation assay (RIPA) buffer supplemented with a protease inhibitor mini-tablet (Roche, Mannheim, Germany). The lysates were cleared by centrifugation at 14,000 rpm for 15 min. Protein concentrations were measured using the Pierce BCA assay kit (Thermo Fisher Scientific, Waltham, MA, USA, #23227).

Equal amounts of protein lysates (20 µg) were separated by sodium dodecyl sulfate-polyacrylamide gel electrophoresis (SDS-PAGE) using Mini-PROTEAN TGX Stain-Free Gels (Bio-Rad, Hercules, CA, USA, #4568096) and transferred to PVDF membranes (Bio-Rad, #10026934) using the Trans-Blot Turbo system (Bio-Rad). Blots were incubated with the following primary antibodies recognizing: β-actin (MAB1501R, Millipore, Burlington, MA, USA, 1:5000), PARP (#9542, 1:1000), XIAP (#2045, 1:1000), Bax (#5023, 1:1000), p21(a (#2947, 1:1000), cleaved caspase 3 (#9661, 1:1000), caspase 3 (#9662, 1:1000), caspase 7 (#9492, 1:1000), and Survivin (#2808, 1:1000) (Cell Signaling Technologies, Danvers, MA, USA), paired with secondary HRP antibody (#405306, BioLegend, San Diego, CA, USA, 1:10,000) for β-actin probed blots, or with secondary HRP antibody (#31458, Invitrogen, 1:10,000) for all other primary antibodies. Signals were developed using Clarity Western ECL Substrate (Bio-Rad, #170-5061) and imaged with ChemiDoc MP Imaging System (Bio-Rad). Band intensities were quantified by densitometry and normalized to β-actin using ImageLab 3.0.1. (Bio-Rad).

### 2.7. Immunofluorescence and Confocal Microscopy

Cells were seeded onto MatTek 35 mm glass bottom microwell dishes (P35GC-0-14-C), treated with SN-38, YM-155, CuET, or the indicated combinations, and incubated for 72 h. Following treatment, cells were fixed with freshly prepared, methanol-free 4% paraformaldehyde in PBS for 20 min at room temperature. Samples were permeabilized for 10 min at room temperature using 1% sodium citrate and 0.1% Triton X-100 in PBS. Cells were then blocked for 60 min in PBS containing 5% bovine serum albumin and 0.3% Triton X-100. Survivin was detected using rabbit monoclonal anti-Survivin antibody 71G4B7 (Cell Signaling Technology) diluted 1:200 in PBS containing 1% bovine serum albumin and 0.3% Triton X-100. Samples were incubated with the primary antibody overnight at 4 °C. Cells were then incubated for 1 h at room temperature in the dark with Alexa Fluor 488-conjugated F(ab′)_2_ goat anti-rabbit IgG secondary antibody (Thermo Fisher Scientific, Waltham, MA, USA, #A-11070) at 2 µg/mL. Nuclei were counterstained for 10 min with Hoechst 33342 (Thermo Fisher Scientific, #62249) at a final concentration of 1 µg/mL.

Fluorescence images were acquired by confocal microscopy on Zeiss LSM880 using identical acquisition settings for all experimental groups within each experiment. Hoechst, Survivin-Alexa Fluor 488, and merged images were collected. Survivin fluorescence was quantified as mean fluorescence intensity after background subtraction using QuPath Bioimage Analysis Software v0.7.0.

### 2.8. Annexin V Flow-Cytometric Analysis of Apoptosis

HT-29 cells were treated with 20 nM SN-38, 20 nM YM-155, or their combination for 72 h. Both the culture supernatant, containing detached or apoptotic cells, and adherent cells released by trypsinization were collected by centrifugation. Cells were washed twice with cold PBS and resuspended in 1× Annexin V binding buffer containing 10 mM HEPES, 140 mM NaCl, and 2.5 mM CaCl_2_ at pH 7.4.

For viability discrimination, cells were first incubated with Blue Fluorescent LIVE/DEAD Fixable Dead Cell Stain (Thermo Fisher Scientific, #L34962). The dye was reconstituted in 50 µL DMSO, and 1 µL was added per 1 mL cell suspension containing approximately 1 × 10^6^ cells. Samples were incubated for 15 min at room temperature, protected from light, and washed twice with PBS. Cells were then adjusted to approximately 1 × 10^6^ cells/mL in Annexin V binding buffer. A 100-µL aliquot containing approximately 1 × 10^5^ cells was transferred to each flow-cytometry tube, supplemented with 5 µL Annexin V-FITC (Biolegend, # 640906), and incubated for 10 min at room temperature in the dark. Following staining, 400 µL Annexin V binding buffer was added, and samples were maintained on ice until acquired on BD LRS Fortessa (Franklin Lakes, NJ, USA).

Unstained, single-stained, and untreated controls were included for compensation and gating. Viable cells were defined as Annexin V-negative/viability dye-negative, early-apoptotic cells as Annexin V-positive/viability dye-negative, and late-apoptotic or membrane-compromised cells as Annexin V-positive/viability dye-positive. The percentages of early- and late-apoptotic cells were quantified using identical gating criteria across treatment groups in FlowJo v10.10.1.

### 2.9. Mice

Six-week-old SCID-beige and C57BL/6 mice of both sexes were purchased from Charles River Laboratories (Montreal, QC, Canada) and maintained under specific pathogen-free conditions.

At eight weeks, SCID-beige mice received 100 μL of HCT116 Parental cells at a final concentration of 5 × 10^7^ cells/mL in PBS, subcutaneously injected into the left flank. When tumors reached approximately 100 mm^3^ (day 10), mice were randomly assigned into the following treatment groups: Control (PBS + DMSO), YM-155 (5 mg/kg/day) administered continuously via slow-release osmotic pumps, CPT-11 (20 mg/kg) administered intraperitoneally (i.p.) every 4 days, or the combination of YM-155 (osmotic pump) + CPT-11 (i.p.). Implantable slow-release osmotic pumps (ALZET) containing YM-155 were implanted in the right flanks of the mice.

Tumor dimensions were measured with an electronic caliper every two or three days, and tumor volumes were calculated using the formula: V (mm^3^) = (L × W^2^)/2, where L and W are the length and width of the tumor. Endpoint tumor volume was defined as 2 cm^3^ according to the FACC guidelines. Mouse weight was recorded every second day.

Eight-week-old C57BL/6 mice received 100 μL of MC-38 cells at a final concentration of 3.2 × 10^6^ cells/mL in PBS, subcutaneously injected between the shoulder blades. Mice were randomized based on body weight into the following treatment groups: untreated, mouse albumin (MSA) + DMSO, CPT-11 (20 mg/kg), CuET (1 mg/kg in MSA), and CPT-11 + CuET. Treatments were administered through i.p. injections beginning on day 6 after tumor implantation, following a schedule of five consecutive treatment days followed by two days of break, repeated for two courses until the study endpoint (day 20). Tumor growth was monitored every second day by measuring width (W) and length (L) with an electronic caliper, and tumor volume (V) was calculated using the formula: (V (mm^3^) = (L × W^2^)/2). Body weights were recorded every second day. Endpoint tumor volume was defined as 2 cm^3^ according to the FACC guidelines. All experimental procedures were approved by the Facility Animal Care Committee of the McGill University Health Centre (Montreal, QC, Canada).

#### Albumin-Based CuET Nanoparticle Synthesis

For *in vivo* experiments, mouse albumin-based CuET nanoparticles (1 mg/mL) were synthesized as previously described [34]. Briefly, diethyldithiocarbamate (Sigma), dissolved in water, was mixed with copper (II) chloride (Sigma), also dissolved in water, at a 2:1 molar ratio. The reaction was carried out in a 5% (*w*/*v*) mouse serum albumin solution (Innovative Research, Novi, MI, USA). The CuET nanoparticles are stable at 4 °C for at least one week. The administered dose corresponded to 1 mg/kg of diethyldithiocarbamate copper complex in a total (i.p.) injection volume of 0.5 mL isotonic salt.

### 2.10. TUNEL Assay

The assay was performed according to the manufacturer’s instructions using an *in situ* apoptosis detection kit (MK500, Takara Bio, Katsuyama, Japan) to assess DNA fragmentation in the HCT116 Parental tumors from SCID-beige mice treated with YM-155 and CPT-11. Briefly, tissue sections were deparaffinized and hydrated through graded ethanol solutions. The slides were washed in PBS and incubated with proteinase K (20 μg/mL) for 15 min. Endogenous peroxidases were quenched by incubating sections with 0.3% H_2_O_2_ at room temperature for 15–30 min. The TUNEL reaction mixture was then applied to label DNA strand breaks with fluorescein-dUTP, and slides were incubated in a humidified chamber at 37 °C for 1 h in the dark. Anti-FITC HRP conjugate was subsequently added and incubated at 37 °C for 30 min. Next, the slides were washed, and DAB substrate was applied for 15 min at room temperature. Slides were then washed again and counterstained with 1.5% methyl green (Sigma). Whole-slide imaging was performed using a Leica Aperio AT Turbo digital pathology scanner at 40X magnification (25 microns/pixel). Representative whole-tumor sections were analyzed using QuPath Bioimage Analysis Software v0.7.0., and TUNEL-positive cells were quantified as a percentage of the total cells from whole sections.

### 2.11. Immunohistochemistry (IHC)

Tumors from treated and control mice were surgically excised at endpoint, fixed in 10% PBS buffered formalin for 48 h, embedded in paraffin, and sectioned at 4 µm of thickness. Hematoxylin and eosin (H&E) staining was performed. Immunostaining was carried out using an automated immunostainer (Discovery XT; Ventana Medical Systems, Tucson, AZ, USA). Tumor tissue sections were stained with anti-Ki67 (#15580, Abcam, Cambridge, UK), anti-cleaved caspase 3 (#9661, Cell Signaling), and anti-Survivin (#PA1-16836, Invitrogen). Slides were scanned using a Leica Aperio AT Turbo digital pathology scanner (Leica Biosystems, Deer Park, IL, USA) at 40X magnification (25 microns/pixel). Ki67-, cleaved caspase-3-, Survivin-, and TUNEL-positive cells were quantified as percentages of total cells from whole sections using consistent analysis settings across treatment groups, in QuPath Bioimage Analysis Software v0.7.0.

For assessment of systemic toxicity, heart, liver, and kidney tissues were collected at endpoint, fixed in 10% PBS buffered formalin, paraffin-embedded, sectioned, and stained with H&E. Sections were examined for treatment-associated alterations in tissue architecture, necrosis, inflammatory infiltration, and other overt morphological abnormalities.

### 2.12. Statistical Analyses

Statistical analyses were performed using the GraphPad Prism 11 software (GraphPad Software, San Diego, CA, USA). One-way ANOVA or two-way ANOVA tests with the indicated multiple-comparison correction were used for comparisons involving one or two independent variables, respectively. The Brown–Forsythe and Welch ANOVA with Dunnett’s T3 multiple-comparison test was used when the variance assumptions were not met. Two-way ANOVA was used to evaluate concentration and time effects in spheroid experiments. For comparisons between two conditions involving a single variable, Welch’s *t*-test or the indicated corrected multiple-comparison test was used. Data are presented as mean ± SD unless otherwise indicated (* *p* < 0.05, ** *p* < 0.01, *** *p* < 0.001 and **** *p* < 0.0001).

## 3. Results

### 3.1. CPT-11 Metabolite SN-38 and YM-155 Synergize to Inhibit Colorectal Cancer Cell Growth

**Figure 1:**
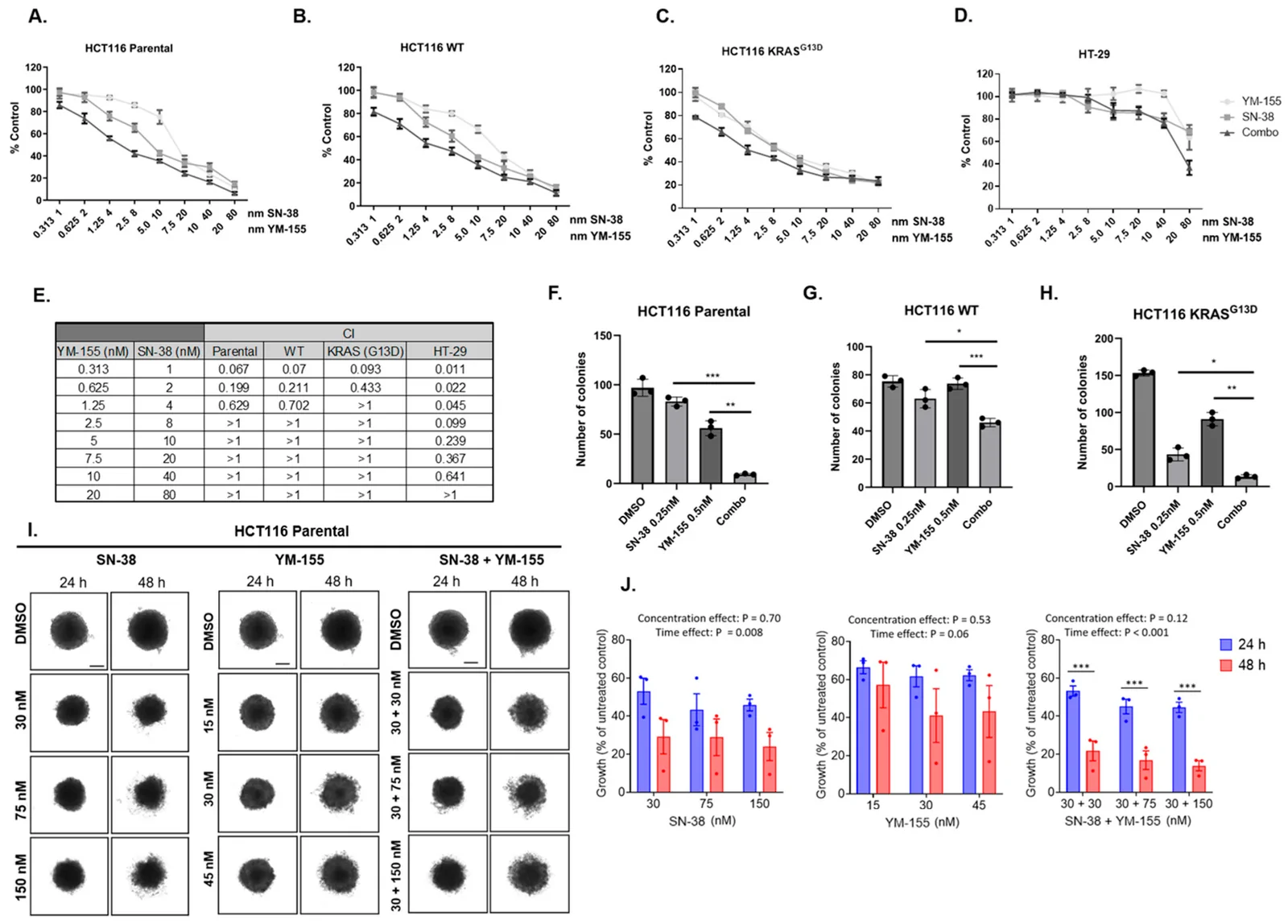
SN-38 and YM-155 synergize to inhibit colorectal cancer cell growth. (**A**–**D**) CRC cell lines (HCT116 Parental, WT, *KRAS*^G13D^, and HT-29) were treated with serial dilutions of YM-155 (0 to 20 nM) or SN-38 (0 to 80 nM) or their combination for 72 h, and cytotoxicity was evaluated by XTT assay. Data represented as mean ± SEM. (**E**) Combination index (CI) of cytotoxicity assay in (**A**–**D**), calculated using Chou-Talalay Method, reported for synergistic combination doses, where CI <1: synergy, CI =1: additive effect, CI >1: antagonism. (**F**–**H**) Colony formation assays were performed after treating CRC cell lines with YM-155 (0.5 nM) or SN-38 (0.25 nM) or their combination for seven consecutive days. Data represent three independent experiments, mean ± SD. Welch’s t test, where * *p* < 0.05, ** *p* < 0.01 and *** *p* < 0.001. (**I**) Representative images of HCT116 Parental spheroids treated with SN-38 (30, 75, or 150 nM), YM-155 (15, 30, or 45 nM), or SN-38 plus YM-155 at the indicated concentrations for 24 or 48 h. Scale bars are 100 µm. (**J**) Quantification of spheroid growth at 24 and 48 h, expressed as a percentage of the DMSO control. Concentration and time effects were assessed as indicated above each graph. Data are mean ± SEM, n = 3 independent replicates, Ordinary two-way ANOVA with concentration and time as factors, followed by Šídák’s multiple comparisons test for 24 h versus 48 h comparisons within each concentration.

To determine whether Survivin inhibition potentiates irinotecan-induced cytotoxicity, we assessed the combined activity of the Survivin inhibitor YM-155 and the active irinotecan metabolite SN-38 on CRC cell viability. HCT116 Parental, WT, *KRAS*^G13D^, and HT-29 cells were treated with increasing concentrations of SN-38 and YM-155, either individually or in combination, and cell viability was measured after 72 h using an XTT assay. In all four cell lines, the combination treatment consistently reduced cell viability more than single-agents at synergistic concentrations, although the magnitude and concentration range of this effect varied among the cell lines (Figure 1A–D). The dose–response curves consistently showed stronger growth inhibition with the combination, indicating a synergistic interaction between SN-38–induced DNA damage and Survivin inhibition.

The synergy between the two agents was assessed by calculating the combination index (CI) using the Chou-Talalay method [41,42]. The analysis identified synergistic interactions (CI < 1) between SN-38 and YM-155 at selected paired concentrations in all four cell lines (Figure 1E). In HCT116 Parental and WT cells, synergism was observed at doses of and below 4 nM of SN-38 and 1.25 nM of YM-155. In HCT116 *KRAS*^G13D^, synergism occurred at doses of and below 2 nM of SN-38 and 0.625 nM of YM-155. In contrast, HT-29 cells exhibited synergy over a broader range, extending to 40 nM SN-38 and 10 nM YM-155 (Figure 1E). Thus, the interaction was concentration- and cell line-dependent rather than uniformly synergistic across the complete dose range.

To further assess the long-term impact of this combination on CRC cell survival, colony formation assays were performed following treatment with YM-155 (0.5 nM), SN-38 (0.25 nM), or their combination for seven consecutive days. While each drug alone produced a cell-line-dependent reduction in clonogenic survival, the combination treatment markedly suppressed colony formation in all three HCT116 cell lines (Figure 1F–H). Quantification revealed a significant reduction in colony number in the combination group compared with either single-agent treatment, indicating that concurrent inhibition of Survivin and topoisomerase I activity effectively impairs the long-term proliferative capacity of CRC cells. In HCT116 Parental and WT cells, SN-38 monotherapy reduced colony growth by approximately 20% compared with the DMSO control. When combined with YM-155, the colony growth was significantly reduced by 80% and 50%, respectively. In HCT116 *KRAS*^G13D^ cells, SN-38 alone achieved about 60% inhibition of colony formation, whereas the combination with YM-155 resulted in approximately 85% inhibition.

The activity of the treatments was additionally evaluated in three-dimensional HCT116 Parental spheroids. Representative images showed progressive disruption of spheroid growth and morphology following exposure to SN-38, YM-155, or their combination for 24 and 48 h (Figure 1I). Quantitative analysis indicated a significant time-dependent reduction in spheroid growth following SN-38 treatment (time effect, *p* = 0.008), without a significant concentration effect over the tested range (*p* = 0.70). Similarly, YM-155 showed no significant concentration effect (*p* = 0.53) and only a borderline time effect (*p* = 0.06). In contrast, combined treatment produced a pronounced time-dependent reduction in spheroid growth (*p* < 0.001), with growth at 48 h significantly lower than at 24 h for each tested combination (Figure 1J). These results indicate that treatment duration was a major determinant of the response in the three-dimensional model, particularly for the combination treatment.

Collectively, these findings demonstrate that Survivin inhibition by YM-155 enhances SN-38–induced cytotoxicity and suppresses long-term clonogenic survival in CRC cells. The three-dimensional spheroid data further support a time-dependent inhibitory effect of the combination under conditions that more closely approximate tumor architecture. Importantly, the observed dose-dependent synergy underscores the potential therapeutic benefit of combining Survivin inhibition with irinotecan-based chemotherapy, particularly at optimally selected concentrations.

### 3.2. SN-38-Induced Survivin Expression in Colorectal Cancer Cells Is Inhibited by YM-155

**Figure 2:**
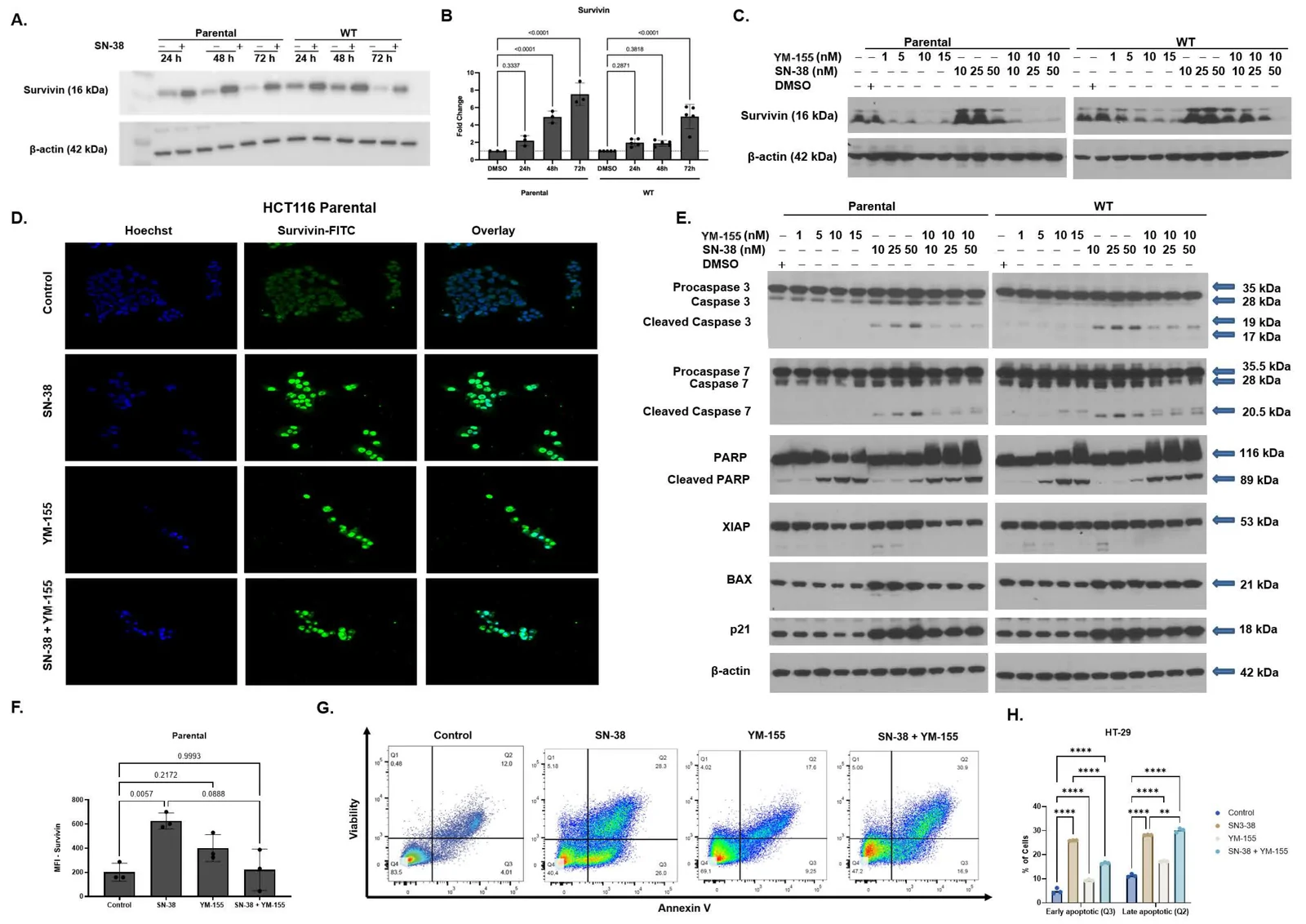
The combination of YM-155 and SN-38 inhibits Survivin and induces caspase-dependent apoptosis in colorectal cancer cells, in a dose-dependent manner. (**A**) Survivin expression in cell lysates from HCT116 Parental and WT treated with SN-38 (10 nM) for 24, 48, and 72 h (Images S1 and S2). (**B**) Densiometric quantification of Survivin expression in cell lysates from HCT116 Parental (n = 3) and WT (n = 5) treated with SN-38 (10 nM) for 24, 48, and 72 h. Survivin expression was normalized to β-actin and expressed as fold change relative to the corresponding control. The dotted line indicates a fold-change value of 1. *p* values are shown. (**C**) Survivin expression in cell lysates from HCT116 Parental and WT treated with SN-38 (10 to 50 nM) and/or YM-155 (1 to 15 nM) for 48 h (Images S3 and S4). (**D**) Representative immunofluorescence images of HCT116 Parental cells treated with DMSO control, SN-38 (20 nM), YM-155 (20 nM), or SN-38 plus YM-155, for 72 h. Nuclei were stained with Hoechst, and Survivin was detected by FITC fluorescence. (**E**) Expression of caspases 3 and 7, PARP, XIAP, BAX, and p21 proteins in cell lysates from HCT116 Parental and WT treated with SN-38 (10 to 50 nM) and/or YM-155 (1 to 15 nM) for 48 h. β-actin served as the loading control (Images S5–S11). (**F**) Survivin mean fluorescence intensity in HCT116 Parental cells under the conditions shown in panel (**D**) (n = 3). Data are presented as mean ± SD. *p*-values obtained using Brown-Forsythe and Welch ANOVA with Dunnett’s T3 multiple comparison. (**G**) Representative Annexin V/viability flow-cytometry plots of HT-29 cells treated with control, SN-38 (20 nM), YM-155 (20 nM), or SN-38 plus YM-155. (**H**) Quantification of early- and late-apoptotic HT-29 cell populations from the analysis shown in panel (**G**). Data are expressed as mean ± SD percentage of cells in the indicated apoptotic quadrants (n = 3). Brown-Forsythe and Welch ANOVA with Dunnett’s T3 multiple comparison, where ** *p* < 0.005, and **** *p* < 0.0001. β-actin served as the loading control for all Western blot analyses.

To investigate the effect of SN-38 on the inhibitor-of-apoptosis protein Survivin, HCT116 Parental and WT cells were treated with 10 nM SN-38 and analyzed by Western blot over 24, 48, and 72 h. SN-38 treatment induced a time-dependent increase in Survivin expression in both cell lines compared with DMSO controls, with the highest protein levels observed at 48 h (Figure 2A). Densitometric analysis confirmed that Survivin expression increased progressively over time, with the highest levels detected at 72 h in both HCT116 Parental and WT cells (Figure 2B). These results suggest that Survivin upregulation represents a cellular survival response to irinotecan-induced stress, potentially limiting apoptotic signaling.

Next, we evaluated the impact of YM-155 (1–15 nM) on Survivin protein expression in HCT116 Parental and WT cells. Western blot analysis revealed that YM-155 treatment markedly reduced Survivin protein levels in both cell lines to near-null levels compared to untreated and DMSO controls. Importantly, the addition of YM-155 inhibited the rise in Survivin levels caused by SN-38, with greater suppression generally observed as the concentration of YM-155 increased (Figure 2C and Figure S1). These findings indicate that YM-155 effectively counteracts SN-38-mediated Survivin upregulation, providing a potential strategy to overcome apoptotic resistance and enhance irinotecan efficacy in CRC cells.

The effect of YM-155 on SN-38-induced Survivin expression was further examined by immunofluorescence in HCT116 Parental cells (Figure 2D). SN-38 treatment significantly increased Survivin-associated fluorescence compared with the control (*p =* 0.0057), whereas YM-155 alone produced a lower Survivin signal than in SN-38 treated cells. The small, non-significant (*p* = 0.2172) increase in Survivin fluorescence in YM-155-treated cells compared to control may reflect cytotoxic-associated loss of adherent cells, as supported by reduced cell density in brightfield images and cell counts (Figure S2), whereas Western blot analysis reflects bulk protein levels. Thus, differences between assays likely reflect methodological and sampling differences.

Furthermore, the combination of SN-38 and YM-155 reduced Survivin expression to a level indistinguishable from control (Figure 2F). Together, the Western blot and immunofluorescence findings support the ability of YM-155 to attenuate SN-38-induced Survivin protein expression.

### 3.3. YM-155 and SN-38 Combination Treatment Is Associated with Apoptotic Signaling and Increased Cell Death

To determine whether Survivin inhibition was associated with enhanced irinotecan-induced apoptosis, we assessed markers of caspase-dependent apoptotic signaling in HCT116 cells treated with SN-38, YM-155, and their combination. Cleavage of caspase-3, caspase-7, and poly ADP-ribose polymerase (PARP) was detected following treatment, consistent with engagement of execution-phase apoptotic signaling (Figure 2E and Figure S3). Although cleaved caspase-3 and cleaved caspase-7 were not consistently as pronounced in the combination treatment as in the SN-38-alone condition, their cleavage remained detectable, indicating that caspase-dependent apoptosis contributes to the treatment response. At the same time, the strong PARP cleavage observed with the combination suggests that caspase-3/-7 activation may not represent the exclusive mechanism underlying the enhanced cytotoxicity. These findings are therefore consistent with the engagement of caspase-dependent apoptotic signaling, while also allowing for the possibility that YM-155 contributes to additional regulated cell-death mechanisms.

These effects were observed in parallel with a decrease in the X-linked inhibitor of apoptosis protein (XIAP) and an increase in the levels of pro-apoptotic Bcl-2-associated X protein (BAX), as compared to controls, consistent with activation of the intrinsic apoptotic pathway. Additionally, cyclin-dependent kinase inhibitor p21 expression was elevated following SN-38 and combination treatment, consistent with activation of cellular stress and DNA damage response pathways that may contribute to growth arrest and apoptotic signaling. Overall, YM-155 enhances SN-38-induced cytotoxicity and is accompanied by changes in several apoptosis-related proteins.

Apoptosis was further evaluated in HT-29 cells by Annexin V/viability staining. Representative flow cytometry plots showed that SN-38, YM-155, and their combination increased apoptotic cell populations relative to the control (Figure 2G). SN-38 and YM-155 treatments resulted in significant increases in the early- and late-apoptotic populations. SN-38 treatment produced a prominent early apoptotic population, whereas the combination produced a shift from the Annexin V-positive/FVD-negative compartment toward the Annexin V-positive/FVD-positive compartment. The total Annexin V-positive fraction supports treatment-associated apoptosis and late-stage cell death, although Annexin V/FVD staining alone cannot determine whether all Q2 cells are late apoptotic rather than secondary-necrotic or otherwise membrane-compromised cells. The combination generated the largest Annexin V-positive/FVD-positive fraction among the tested conditions, consistent with increased progression toward a late-stage cell-death phenotype (Figure 2H).

Collectively, these data demonstrate that YM-155 suppresses SN-38-induced Survivin accumulation and that combination treatment is associated with apoptotic and late-stage cell-death phenotypes in CRC cells. The Western blot, immunofluorescence, and flow cytometry findings support a model in which Survivin inhibition facilitates the progression of SN-38-treated cells toward apoptosis, albeit without excluding contributions from caspase-independent apoptosis or other regulated cell-death mechanisms.

### 3.4. In Vivo, the Combination of YM-155 with CPT-11 Inhibits Colorectal Cancer Growth and Induces Apoptosis

**Figure 3:**
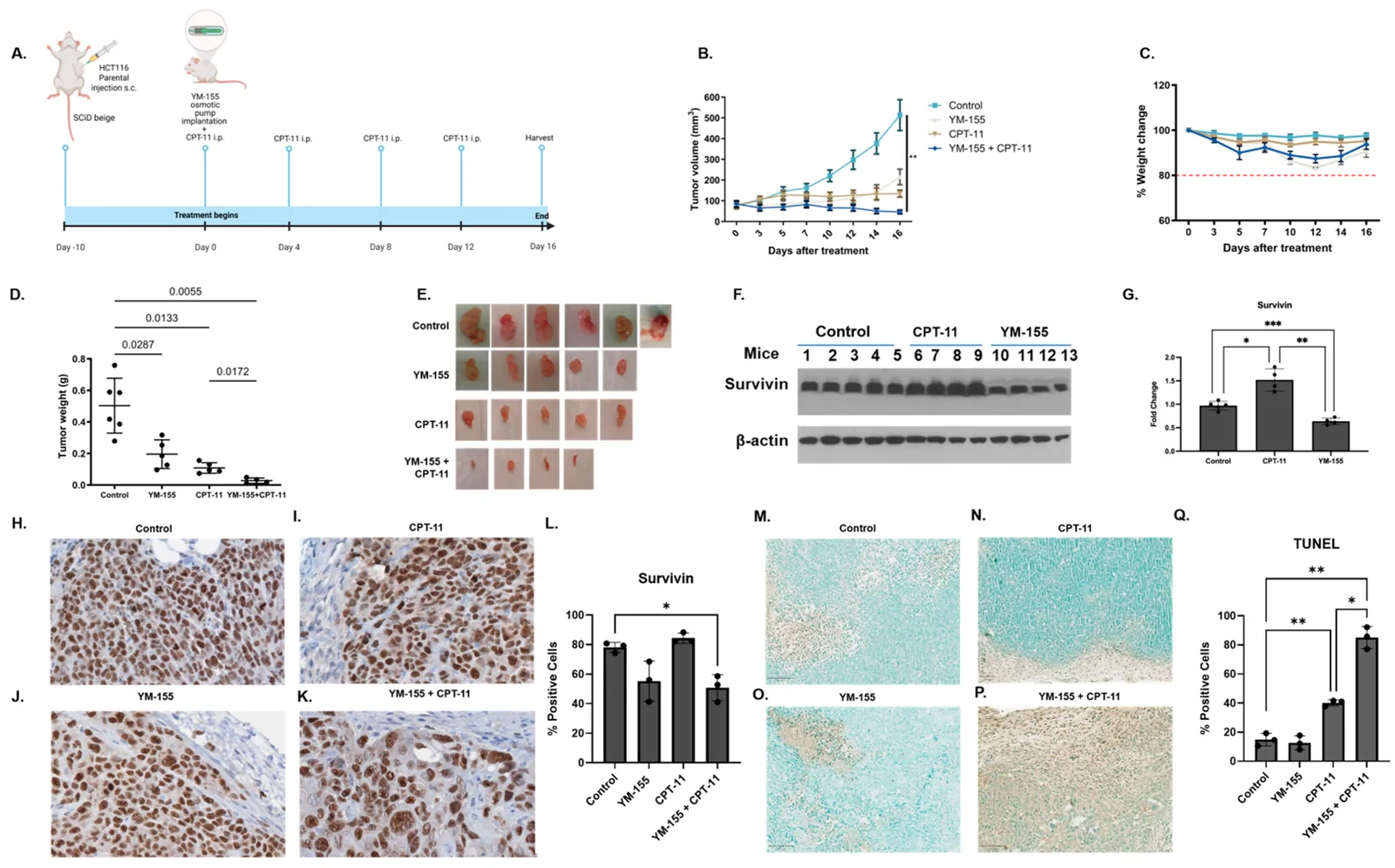
YM-155 enhances the antitumor activity of CPT-11 *in vivo* and promotes apoptosis in HCT116 xenografts. (**A**) *In vivo* experimental design showing treatment groups: Control (n = 6), YM-155 (n = 5), CPT-11 (n = 5), and YM-155 + CPT-11 (n = 4). (**B**) Tumor growth inhibition in HCT116 Parental xenografts treated with YM-155 (5 mg/kg/day), CPT-11 (20 mg/kg), and their combination in an ectopic CRC model in SCID beige mice. Data are shown as mean ± SEM. Dunnett’s Two-Way ANOVA, where ** *p* < 0.005. (**C**) Body-weight changes in SCID beige mice during treatment with CPT-11, YM-155, their combination, and vehicle control. Data are shown as mean ± SEM. The red dotted line indicates 80%, or a biologically significant weight reduction of 20%. (**D**) Tumor weights of HCT116 Parental xenografts at the study endpoint in each treatment group. Data are presented as mean ± SD. *p*-values obtained using Brown-Forsythe and Welch ANOVA with Dunnett’s T3 multiple comparison. (**E**) Representative images of excised HCT116 Parental tumors collected from SCID beige mice at endpoint or day 16 of treatment. (**F**) Western blot analysis of Survivin protein levels in the tumor lysates from mice treated with CPT-11, YM-155, and control (Images S12 and S13). (**G**) Densiometric quantification of Survivin expression from the Western blot analysis in panel (**F**) (n = 4 to 5 per group). Data are presented as mean ± SD. Welch’s *t*-test, where * *p* < 0.05, ** *p* < 0.005, and *** *p* < 0.0005. (**H**–**K**) Representative IHC staining of Survivin protein in HCT116 Parental tumors from SCID beige mice treated with YM-155, CPT-11, their combination, or control. (**L**) Quantification of Survivin-positive tumor cells in the groups shown in panels (**H**–**K**) (n = 3). Data are presented as mean ± SD. Brown-Forsythe and Welch ANOVA with Dunnett’s T3 multiple comparison, where * *p* < 0.05. (**M**–**P**) Representative TUNEL staining to assess apoptosis in HCT116 Parental tumors of SCID beige mice treated with YM-155, CPT-11, their combination, or control. Scale bar is 100 µm. (**Q**) Quantification of TUNEL-positive cells in the groups shown in panels (**M**–**P**) (n = 3). Data are presented as mean ± SD. Brown-Forsythe and Welch ANOVA with Dunnett’s T3 multiple comparison, where * *p* < 0.05, and ** *p* < 0.005.

We next evaluated the therapeutic effects of YM-155, CPT-11, and their combination, in an HCT116 Parental xenograft model in SCID beige mice, to determine whether Survivin inhibition enhances irinotecan efficacy *in vivo*. As outlined in Figure 3A, mice bearing established tumors (~100 mm^3^) received YM-155 (5 mg/kg/day), CPT-11 (20 mg/kg), or the combination, and tumor growth was monitored throughout the treatment period. YM-155 was administered continuously following osmotic pump implantation, whereas CPT-11 was administered intraperitoneally on days 4, 8, and 12. Tumors were harvested on day 16.

Consistent with the *in vitro* findings, the combination therapy markedly suppressed tumor growth compared with either monotherapy. While tumors in the control group continued to grow progressively, treatment with YM-155 or CPT-11 alone produced a moderate reduction in tumor growth. The combined treatment produced the greatest and most sustained suppression of tumor growth during the treatment period (Figure 3B). Importantly, no biologically significant differences in body weight were observed among treatment groups with none of the mice losing 20% or more of their initial body weight, indicating that the combination regimen was well tolerated and did not induce overt systemic toxicity (Figure 3C).

At the experimental endpoint, tumor weights were reduced by all three treatment regimens relative to the control group. The combination group exhibited the lowest tumor weights, with a significant reduction relative to the control group and CPT-11 monotherapy (Figure 3D). Representative images of the excised tumors were consistent with the tumor-weight measurements and showed the smallest tumors in the combination group (Figure 3E). These results demonstrate that the addition of YM-155 increases the antitumor effect of CPT-11 in this HCT116 xenograft model.

To investigate the molecular effects of the treatments on Survivin expression, Western blot analysis was performed on tumor lysates. CPT-11-treated tumors exhibited increased Survivin protein abundance compared with control tumors, whereas YM-155 treatment reduced Survivin expression relative to both the control and CPT-11 groups, confirmed by densiometric analysis (Figure 3F,G). These *in vivo* findings are consistent with the *in vitro* results and suggest that CPT-11 induces a Survivin-associated stress response that can be suppressed by YM-155.

IHC staining further confirmed these findings. Survivin expression was prominent in control and CPT-11-treated tumors, whereas tumors of mice receiving the YM-155 and CPT-11 combination showed a reduced overall proportion of Survivin-positive cells, despite their markedly enlarged morphology which may render difficult to appreciate the difference in staining (Figure 3H–K). Quantification showed a significant reduction in the percentage of Survivin-positive cells in the combination group relative to the control group (Figure 3L).

To determine whether the observed tumor growth inhibition was associated with increased apoptotic cell death, TUNEL staining was performed on tumor sections. The combination treatment group showed a pronounced increase in apoptotic cells, indicating enhanced apoptosis compared with single agent treatment (Figure 3M–Q and Figure S4). Thus, the inhibition of Survivin with YM-155 significantly enhanced the antitumor efficacy of CPT-11 *in vivo*, leading to reduced tumor growth and increased apoptosis in HCT116 Parental xenografts. These findings support the therapeutic benefit of targeting Survivin to overcome resistance to irinotecan-based chemotherapy.

### 3.5. CuET Retains Cytotoxic Activity in TP53-Deficient Colorectal Cancer Cells and Enhances SN-38-Mediated Growth Inhibition

Although our earlier findings show that Survivin inhibition amplifies SN-38-induced apoptosis in *TP53*-proficient CRC cells, *TP53* mutations are present in roughly 50–60% of CRC cases and are closely linked to elevated Survivin expression and resistance to DNA-damaging chemotherapies such as irinotecan. We therefore examined whether CuET—the active metabolite of disulfiram, known to induce proteotoxic stress and trigger apoptosis independently of p53 signaling—could overcome SN-38 resistance in *TP53*-deficient CRC models through the modulation of Survivin expression.

**Figure 4:**
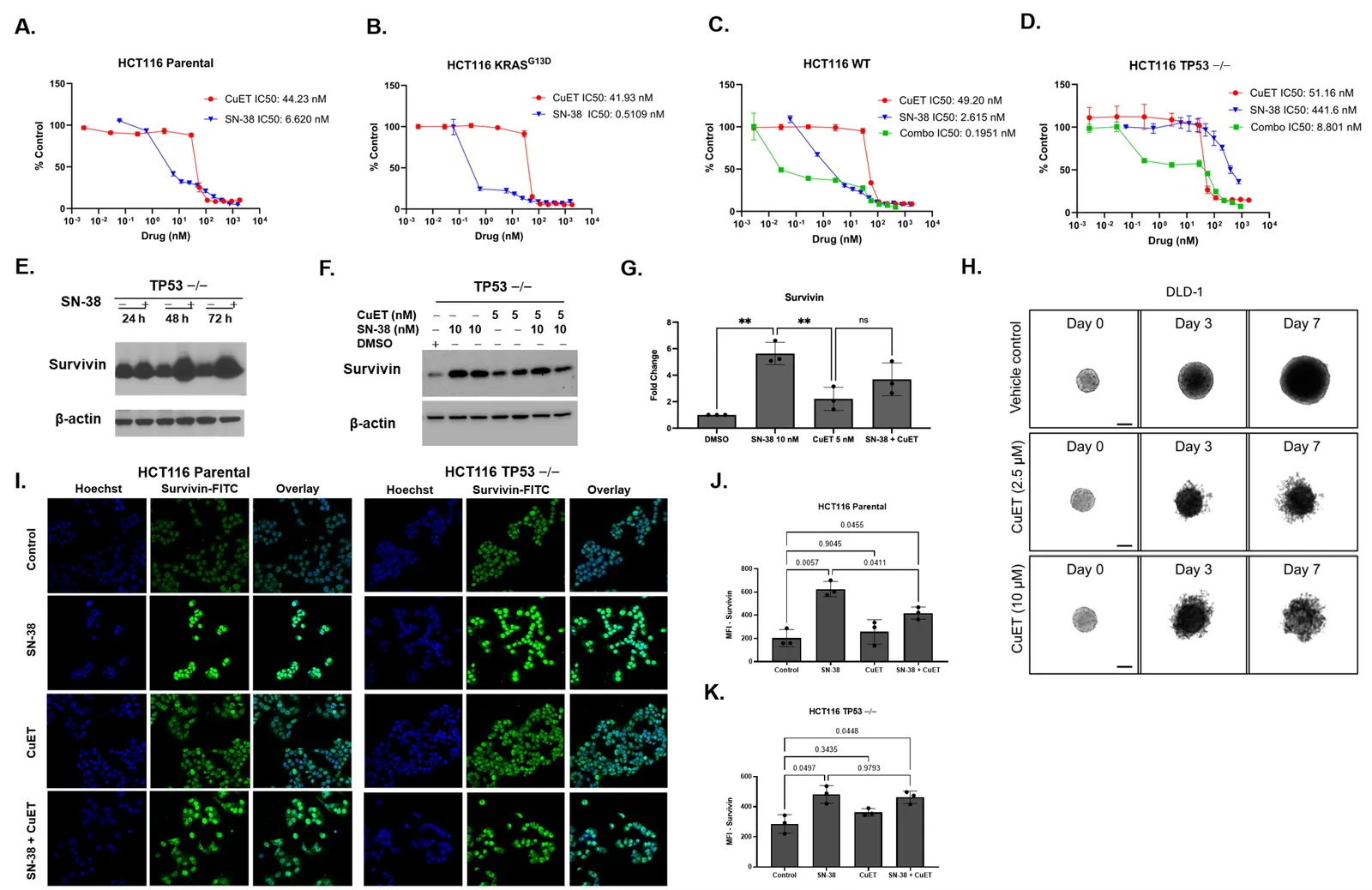
CuET retains cytotoxic potency in *TP53*-deficient colorectal cancer cells that are resistant to SN-38. (**A**–**D**) Dose–response curves showing the cell viability of HCT116 Parental, HCT116 *KRAS*^G13D^, HCT116 WT, and HCT116 *TP53*^−/−^ treated for 72 h with serial dilutions of CuET (0 nM to 1.8μM), SN-38 (0 nM to 1.5 μM), or their combination. CuET retains strong cytotoxic activity in the absence of *TP53*, whereas SN-38 shows dramatically reduced efficacy consistent with *TP53*-dependent resistance. Cytotoxicity was measured using the Sulforhodamine B assay and cell viability is presented as percentage of control, means ± SD. IC_50_ values for each treatment are indicated. (**E**) Western blot analysis of Survivin expression in HCT116 *TP53*^−/−^ cells treated with SN-38 (10 nM) for 24, 48, and 72 h (Image S14). (**F**) Western blot analysis of Survivin expression in HCT116 *TP53*^−/−^ cells treated with CuET (5 nM), SN-38 (10 nM) or the combination for 48 h (Images S15 and S16). β-actin served as a loading control. (**G**) Densitometric quantification of Survivin expression under the conditions shown in panel (**F**). Survivin abundance was normalized to β-actin and expressed as fold change relative to the DMSO control (n = 3). Data are presented as mean ± SD. Welch’s *t*-test, where ** *p* < 0.005. (**H**) Representative bright-field images of DLD-1 spheroids treated with vehicle, CuET (2.5 µM), or CuET (10 µM) and monitored on days 0, 3, and 7. Scale bar is 100 µm. CuET treatment impaired spheroid expansion and disrupted spheroid morphology in a concentration-dependent manner. (**I**) Representative immunofluorescence images of HCT116 Parental and HCT116 TP53^−/−^ cells treated with DMSO control, SN-38 (20 nM), CuET (100 nM), or SN-38 plus CuET. Nuclei were stained with Hoechst, Survivin was detected using FITC fluorescence, and merged images are shown in the overlay columns. (**J**,**K**) Quantification of Survivin mean fluorescence intensity in HCT116 Parental and HCT116 TP53^−/−^ cells under the conditions shown in panel (**I**) (n = 3). Data are presented as mean ± SD. *p*-values obtained using Brown-Forsythe and Welch ANOVA with Dunnett’s T3 multiple comparison. β-actin served as the loading control for all Western blot analyses.

We first compared the cytotoxic effects of CuET and SN-38 using dose–response analyses across multiple HCT116 cell models. In *TP53*-proficient cell lines, including HCT116 Parental, HCT116 *KRAS*^G13D^, and HCT116 WT cells, SN-38 demonstrated strong cytotoxic activity following 72 h of treatment, with IC_50_ values of 6.62 nM for Parental cells, 0.51 nM for *KRAS*^G13D^ cells, and 2.62 nM for WT cells, reflecting high sensitivity to SN-38 in the presence of functional p53 (Figure 4A–C). In contrast, HCT116 *TP53*^−/−^ cells showed markedly reduced sensitivity to SN-38, with a substantially elevated IC_50_ value of 441.6 nM, consistent with the well-established *TP53*-associated resistance to irinotecan-derived therapy (Figure 4D).

We next evaluated whether the cytotoxic activity of CuET is affected by *TP53* status. Across all tested cell lines, CuET exhibited consistent potency, with similar IC_50_ values of 44.23 nM in HCT116 Parental cells, 41.93 nM in HCT116 *KRAS*^G13D^ cells, 49.20 nM in HCT116 WT cells, and 51.16 nM in HCT116 *TP53*^−/−^ cells (Figure 4A–D). These results indicate that, unlike SN-38, CuET maintains its cytotoxic efficacy independently of *TP53* status.

Furthermore, combining CuET with SN-38 enhanced cytotoxicity in *TP53*-proficient cells. In HCT116 WT cells, the combination produced a markedly lower IC_50_ value (0.195 nM) than single agents (Figure 4C). In *TP53*-deficient cells, the combination treatment also improved growth inhibition relative to SN-38 monotherapy, reducing the IC_50_ to 8.80 nM. These results suggest that CuET can partially overcome SN-38 resistance in the absence of functional p53 (Figure 4D). Although this value remained higher than the combination IC_50_ observed in HCT116 WT cells, it represented a substantial restoration of sensitivity compared with SN-38 alone. These results suggest that CuET can partially overcome SN-38 resistance in the absence of functional p53.

Given that Survivin is frequently overexpressed in *TP53*-mutant CRCs and contributes to chemoresistance, we next examined whether its expression is altered in *TP53*-deficient cells following SN-38 and CuET treatment. Western blot analysis demonstrated that SN-38 alone induced a time-dependent increase in Survivin expression in HCT116 *TP53*^−/−^ cells, with progressively elevated expression at 24, 48, and 72 h (Figure 4E). In contrast, CuET treatment induced Survivin expression at levels above the DMSO control but markedly lesser than SN-38. Notably, combined treatment with CuET and SN-38 reduced Survivin expression relative to SN-38 alone (Figure 4F,G), indicating that CuET partially counteracts the SN-38-induced Survivin upregulation.

These findings were further validated by immunofluorescence in HCT116 Parental and HCT116 *TP53*^−^^/−^ cells. In both cell lines, SN-38 increased Survivin-associated fluorescence relative to the corresponding control, whereas CuET alone produced a lower signal. Combined SN-38 and CuET treatment reduced the Survivin signal relative to SN-38 alone, although the magnitude and statistical significance of this reduction differed between the two cell models (Figure 4I–K). These results are consistent with the Western blot analysis and support the ability of CuET to attenuate SN-38-induced Survivin accumulation in both *TP53*-proficient and -deficient cells.

The activity of CuET was also evaluated in three-dimensional *TP53*-mutated DLD-1 spheroids. Vehicle-treated spheroids increased substantially in size between days 0 and 7, whereas CuET-treated spheroids showed slowed growth and progressive disruption of spheroid architecture. This effect was more pronounced at 10 µM than at 2.5 µM CuET, indicating concentration-dependent inhibition of three-dimensional tumor growth (Figure 4H).

Together, these findings demonstrate that CuET retains strong cytotoxic activity independently of *TP53* status and can partially restore SN-38 sensitivity in *TP53*-deficient CRC cells, potentially by suppressing Survivin-mediated survival signaling.

### 3.6. CuET Enhances the Antitumor Efficacy of CPT-11 and Promotes Apoptosis in a Syngeneic Trp53-Mutated Colorectal Cancer Model

**Figure 5:**
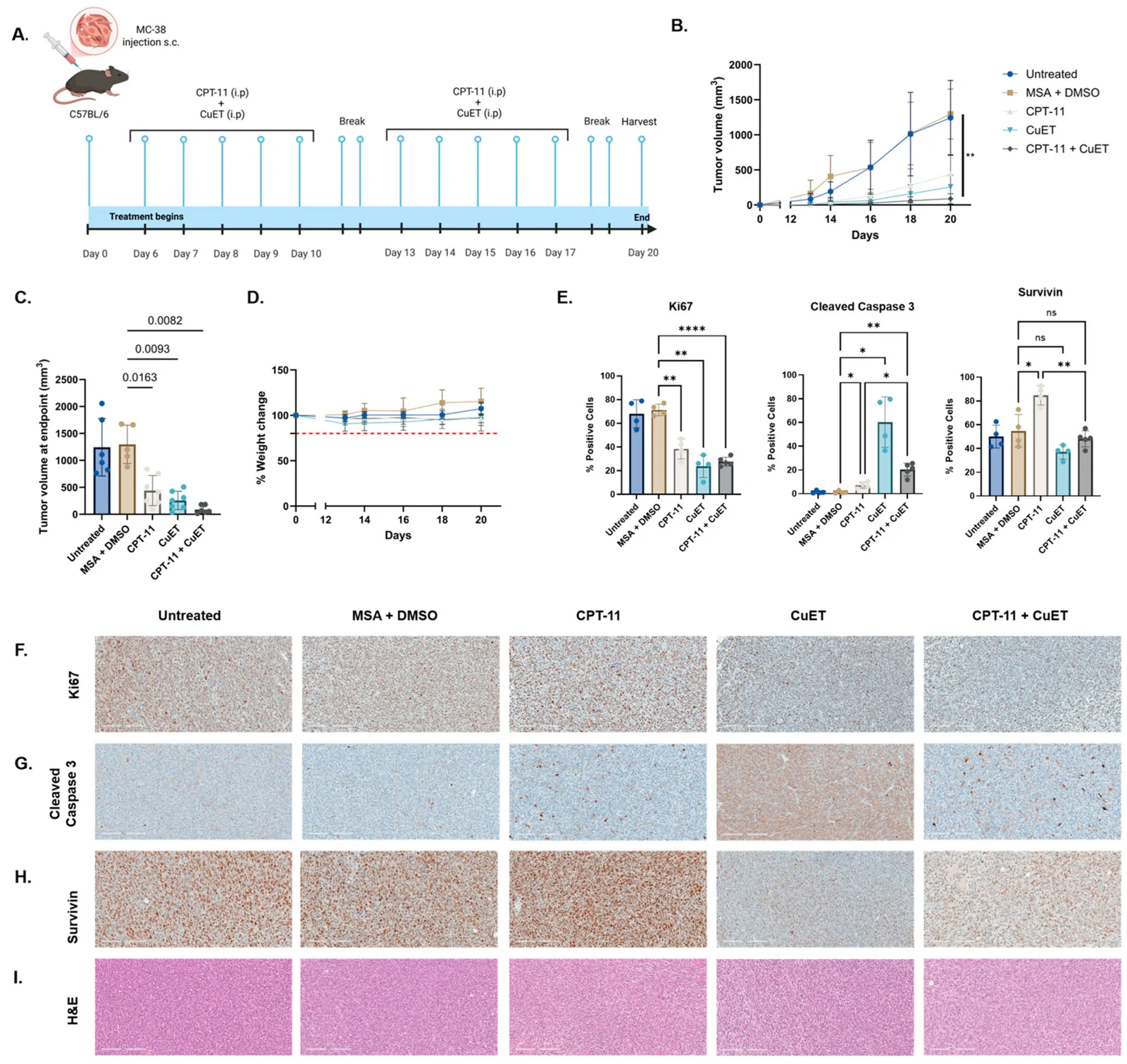
CuET enhances CPT-11 antitumor activity, suppresses Survivin, and induces apoptosis in a syngeneic, *Trp53*-mutant, MC-38 colorectal cancer model. (**A**) *In vivo* experimental design where Untreated (n = 6), MSA + DMSO (n = 5), CPT-11 (n = 7), CuET (n = 7), and CPT-11 + CuET (n = 6). (**B**) MC-38 tumor growth kinetics measured over 20 days in C57BL/6 mice treated with CuET (1 mg/kg/day), CPT-11 (20 mg/kg/day), the combination, or control treatments in a syngeneic ectopic CRC model. Data are presented as mean ± SEM. Two-Way ANOVA with Sidak’s multiple comparison test, (** *p* < 0.005). (**C**) MC-38 tumor volume at day 20 (or endpoint) for each treatment group. Data are shown as mean ± SD. *p*-values were determined using Brown-Forsythe and Welch ANOVA with Dunnett’s T3 multiple comparisons test. (**D**) Body weight changes in C57BL/6 mice during treatment with CuET, CPT-11, the combination, and controls. Data represented as mean ± SEM. The red dotted line marks 80% of initial body weight or a biologically significant weight reduction of 20%. (**E**) Quantification of Ki67-, cleaved caspase-3-, and Survivin-positive cells in MC-38 tumor sections from mice treated with CuET (n = 4), CPT-11 (n = 4), the combination (n = 5), and controls (n = 4). Data are presented as mean ± SD. Brown-Forsythe and Welch ANOVA with Dunnett’s T3 multiple comparison, where * *p* < 0.05, ** *p* < 0.005, and **** *p* < 0.0001. (**F**–**I**) Representative IHC staining for Ki67, cleaved caspase-3, Survivin, and H&E, of the MC-38 tumor tissue from mice treated with CuET, CPT-11, the combination, and controls. Images captured at 20X magnification, scale bar of 200 µm.

Next, we assessed the therapeutic efficacy of combining CuET with CPT-11 in an immunocompetent and *Trp53*-mutated CRC model, using syngeneic MC-38 cells in C57BL/6 mice implanted in C57BL/6 mice. MC-38 cells, which exhibit a very high mutational burden, are representative of human hypermutated CRCs. They also carry two heterozygous *Trp53* missense mutations (G242V and S258I), that result in loss of p53 function [43]. The experimental design is illustrated in Figure 5A. Mice bearing established MC-38 tumors (~100 mm^3^) were treated for 20 days with CuET (1 mg/kg/day), CPT-11 (20 mg/kg/day), the combination of both agents, or control treatments. Tumor growth kinetics revealed that both untreated and vehicle-treated (MSA + DMSO) mice experienced rapid tumor progression, whereas single-agent CPT-11 or CuET produced a noticeable delay in tumor growth. Notably, the CPT-11 + CuET combination achieved the most pronounced and sustained tumor growth inhibition throughout the treatment period, resulting in significantly smaller tumors compared with control groups (*p* < 0.005, Figure 5B). At day 20 (or endpoint), tumors from mice treated with the CPT-11 + CuET combination were significantly smaller than those from untreated or vehicle-treated groups (*p* = 0.0082), as well as smaller than tumors from mice receiving single-agent CPT-11 (*p* = 0.0163) and CuET (*p* = 0.0093) treatments (Figure 5C). These findings demonstrate that CuET enhances the antitumor efficacy of CPT-11 *in vivo*.

Importantly, no significant treatment-related toxicity was observed: body weight remained stable across all groups and never approached the threshold for biologically significant weight loss (20%), indicating that the combination therapy is well tolerated *in vivo* (Figure 5D). Histopathological examination of H&E-stained heart, liver, and kidney sections revealed no overt treatment-associated morphological abnormalities in mice receiving CPT-11, CuET, or the combination compared with untreated and vehicle controls. Overall tissue architecture was preserved, with no obvious evidence of extensive necrosis, inflammatory infiltration, or major organ damage, supporting the absence of marked systemic toxicity under the experimental conditions (Figure S5).

IHC analysis of tumor sections stained for the proliferation marker Ki67 showed reduced proliferative activity in tumors from mice treated with either CPT-11 or CuET, with the lowest Ki67 levels observed in the CuET and combination-treated groups (Figure 5E,F). These findings indicate a marked suppression of tumor cell proliferation following combined therapy.

To assess apoptotic signaling, we stained tumor sections for cleaved caspase-3, a hallmark of execution-phase apoptosis. CuET monotherapy produced the highest proportion of cleaved caspase-3-positive cells, whereas CPT-11 induced a more moderate increase compared with the control groups. The combination group did not show a further increase over CuET alone but displayed higher cleaved caspase-3 staining than the CPT-11 monotherapy group (Figure 5E,G).

Survivin expression was also evaluated in tumor tissue. Strong Survivin staining was detected in untreated, vehicle-treated, and CPT-11-treated tumors. Notably, CuET-treated tumors showed the most pronounced reduction in Survivin expression. Tumors from the CPT-11 + CuET combination group exhibited lower Survivin levels than those from CPT-11 but not significantly different from the untreated or vehicle controls, indicating that CuET attenuated, but did not reduce Survivin expression below basal control levels when combined with CPT-11. These findings suggest that CuET suppresses Survivin expression *in vivo* and may contribute to the enhanced apoptotic response seen with the combination therapy. However, CuET does not fully counteract the induction of Survivin associated with CPT-11 treatment (Figure 5E,H). Finally, H&E staining revealed the overall tumor architecture, showing increased areas of cellular damage and reduced tumor density in the combination-treated group (Figure 5I).

Overall, these results demonstrate that CuET markedly enhances the therapeutic efficacy of CPT-11 in a *Trp53*-mutated CRC model, leading to reduced tumor growth, decreased proliferation, suppression of Survivin expression, and increased caspase-dependent apoptosis. These findings support the potential of CuET-based combination strategies to improve irinotecan efficacy in CRC.

## 4. Discussion

Resistance to DNA-damaging chemotherapy remains a major obstacle in the treatment of mCRC. *TP53* mutations—present in roughly half of all CRC cases—disrupt stress-induced apoptotic signaling and contribute to resistance against irinotecan-based therapies. This study examined the therapeutic potential of combining irinotecan with either direct Survivin suppression or a complementary p53-independent stress-inducing strategy. We identify Survivin-mediated suppression of apoptosis as a key mechanism limiting irinotecan efficacy and demonstrate that pharmacologic targeting of Survivin attenuates this adaptive response and promotes treatment-associated cell death and apoptotic signaling in CRC models. Specifically, we show that YM-155 augments irinotecan-mediated CRC growth inhibition and is associated with increased apoptotic cell populations by suppressing Survivin expression, while the disulfiram metabolite CuET maintains potent cytotoxic activity in *TP53*-deficient cells and further enhances irinotecan efficacy *in vivo*. Together, these results identify treatment-associated Survivin induction as a potentially important adaptive response and support two mechanistically distinct approaches for overcoming it.

A key finding of this study is that irinotecan and its active metabolite SN-38 increase Survivin expression, suggesting that activation of anti-apoptotic pathways represents an adaptive survival response to treatment. Survivin, encoded by *BIRC5*, is a member of the inhibitor of apoptosis protein (IAP) family that suppresses caspase activation and contributes to mitotic progression. The time-dependent accumulation of Survivin following SN-38 exposure was observed in both *TP53*-proficient and -deficient cells, indicating that this response is not restricted to cells with functional p53. This effect was also observed *in vivo*, where CPT-11-treated xenografts exhibited increased Survivin abundance relative to control tumors. The consistency of this response across two-dimensional cultures and tumor tissue supports its biological relevance in establishing Survivin induction as an adaptive resistance mechanism to irinotecan treatment.

Previous studies have shown that Survivin expression correlates with poor prognosis and therapeutic resistance in CRC and multiple other malignancies. Altieri and colleagues established that Survivin acts as a central regulator that integrates cell division, stress-response pathways, and apoptotic signaling while clinical analyses have demonstrated that elevated Survivin levels predict reduced survival following colorectal tumor resection [12,44,45,46,47,48]. Our findings extend these observations by showing that irinotecan treatment itself promotes Survivin accumulation, suggesting that chemotherapy-induced DNA damage may reinforce tumor survival through the activation of this anti-apoptotic pathway.

Survivin represents a broader convergence point for oncogenic signaling beyond the p53–*BIRC5* axis. Zhang et al. demonstrated that YAP activation in Lkb1-deficient lung adenocarcinoma promotes malignant progression through downstream regulation of Survivin, supporting a role for Survivin as an effector of Hippo/YAP-mediated oncogenic signaling [49]. Survivin therefore integrates resistance-associated pathways involving *TP53* dysfunction, *KRAS*-driven adaptive signaling, and YAP/TEAD activation, all of which can promote epithelial proliferation and resistance to cytotoxic or targeted therapies. Mukhopadhyay et al. similarly identified the YAP/TAZ/TEAD pathway as a resistance-associated survival program in *KRAS*-mutant cancers [50]. These observations suggest that suppression of Survivin may disrupt a common downstream survival node shared by genetically distinct CRC subtypes and may therefore contribute to the activity of YM-155- or CuET-based combination strategies.

Importantly, pharmacologic suppression of Survivin with YM-155 enhanced treatment-associated apoptotic signaling and markedly increased irinotecan efficacy. YM-155 reduced Survivin protein levels and synergized with SN-38 to suppress CRC cell viability and clonogenic growth. Our findings indicate that the interaction between YM-155 and SN-38 is concentration- and cell line-dependent, emphasizing the importance of dose selection and tumor context.

Mechanistically, Survivin inhibition was associated with increased BAX expression, reduced XIAP levels, and cleavage of caspase-3, caspase-7, and PARP. Annexin V analysis further confirmed increased apoptotic populations, with combined treatment producing a prominent late-apoptotic fraction. These findings align with earlier studies showing that Survivin counteracts chemotherapy-induced apoptosis by restraining caspase activation and stabilizing microtubule assembly and mitotic progression [23,51,52,53]. Together, these findings support a model in which SN-38-induced DNA damage triggers a compensatory increase in Survivin, limiting apoptosis and contributing to chemoresistance, while YM-155-mediated Survivin suppression enhances cytotoxicity through apoptotic signaling alongside potentially additional regulated cell-death mechanisms.

The growth inhibitory activity of the YM-155 and SN-38 combination was also observed in three-dimensional HCT116 spheroids, in which combined treatment progressively impaired spheroid growth and morphology. The pronounced difference between 24 and 48h suggests that treatment duration was a major determinant of response in this model. Because three-dimensional spheroids incorporate cell–cell interactions and diffusion constraints that are absent from monolayer cultures, these results provide additional support for the activity of the combination under more complex growth conditions. *In vivo*, YM-155 plus CPT-11 produced the greatest reduction in HCT116 xenograft burden and the highest proportion of apoptotic cells, further supporting the therapeutic value of combining direct Survivin suppression with irinotecan.

Although Survivin has long been considered an appealing therapeutic target, clinical translation of Survivin inhibitors has proven challenging. Phase I studies showed that YM-155 could be safely administered and achieved pharmacodynamic suppression of Survivin in tumor tissues, with manageable toxicity profiles [30,31]. However, subsequent phase II trials reported only modest objective response rates across multiple tumor types [32,54,55]. Several factors may account for these limited clinical outcomes, including insufficient drug exposure, tumor heterogeneity, redundant anti-apoptotic signaling, and incomplete suppression of functionally distinct Survivin pools.

Resistance to Survivin-targeted therapy may arise through inadequate drug uptake or increased efflux, incomplete suppression of Survivin isoforms or subcellular pools, compensatory activation of XIAP- and BCL-2-family proteins, and re-expression of *BIRC5* through NF-κB, STAT3, PI3K/AKT/mTOR, or YAP/TEAD signaling [56,57,58,59]. Non-coding RNA networks may further contribute to CRC cell proliferation, apoptosis, invasion, and migration, as illustrated by reciprocal regulation between linc01615 and miR-491-5p [60]. These overlapping resistance mechanisms help explain why direct Survivin inhibition may be more effective in combination with cytotoxic therapy than as a standalone treatment and provide the rationale for evaluating complementary agents that activate additional cell-death pathways.

Because *TP53* mutations are strongly associated with irinotecan resistance, we next investigated whether apoptosis and irinotecan sensitivity could be restored through p53-independent mechanisms. CuET, the active copper-containing metabolite of disulfiram, targets the p97–NPL4 complex and disrupts protein homeostasis, resulting in proteotoxic stress [34]. This mechanism is fundamentally distinct from DNA-damage-based chemotherapy and operates independently of p53 signaling [35,61]. CuET was therefore evaluated not as a direct substitute for YM-155, but as a mechanistically broader strategy capable of inducing p53-independent cytotoxic stress while also attenuating Survivin-associated survival signaling.

Consistent with this rationale, CuET maintained robust cytotoxic potency across CRC cell lines regardless of *TP53* status, whereas sensitivity to SN-38 was markedly reduced in *TP53*-deficient cells. Notably, CuET partially suppressed SN-38-induced Survivin expression and significantly enhanced SN-38 cytotoxicity in *TP53*-knockout cells, suggesting that proteotoxic stress may bypass p53-dependent apoptotic checkpoints. These findings are consistent with recent studies showing that disulfiram/copper can impair survival pathways such as NF-κB signaling, which regulates transcription of anti-apoptotic genes including *BIRC5*, *XIAP*, and *BCL2* [62,63,64]. Thus, the CuET-associated reduction in Survivin is interpreted as one component of a broader response involving proteotoxic stress and disruption of multiple survival pathways that support chemoresistant tumor cells. The inhibition of DLD-1 spheroid expansion and disruption of spheroid architecture further demonstrate that CuET remains active in a three-dimensional, *TP53*-mutated, CRC model.

The therapeutic relevance of this strategy was confirmed in an immunocompetent syngeneic CRC model, where CuET significantly potentiated CPT-11 activity in tumors harboring *Trp53* mutations. The combination therapy produced superior tumor growth inhibition, reduced tumor cell proliferation, and attenuated the increase in Survivin expression associated with CPT-11 treatment. Importantly, Survivin expression was lowest following CuET monotherapy, whereas the combination reduced Survivin relative to CPT-11 but not below that of untreated and vehicle-control levels, indicating that the addition of CuET to CPT-11 partially attenuates the Survivin induction observed *in vivo*.

In the immunocompetent model used, the observed antitumor effect may reflect not only tumor-cell-intrinsic cytotoxicity but also changes in tumor–immune interactions. In our previous work using the same albumin-CuET nanoparticle formulation, CuET increased expression of the stress-inducible NKG2D ligands MICA and MICB on cancer cells and enhanced their recognition by NKG2D-expressing immune effector cells [65]. These findings raise the possibility that immune-mediated mechanisms contribute to CuET activity in the present model. However, immune-cell composition, NKG2D-dependent responses, cytokine production, and potential effects of CPT-11, CuET, and their combination, on normal immune or intestinal cells were not evaluated in the present study. The potential immune contribution of the CuET plus CPT-11 combination therefore remains an important subject for future investigation.

**Figure 6:**
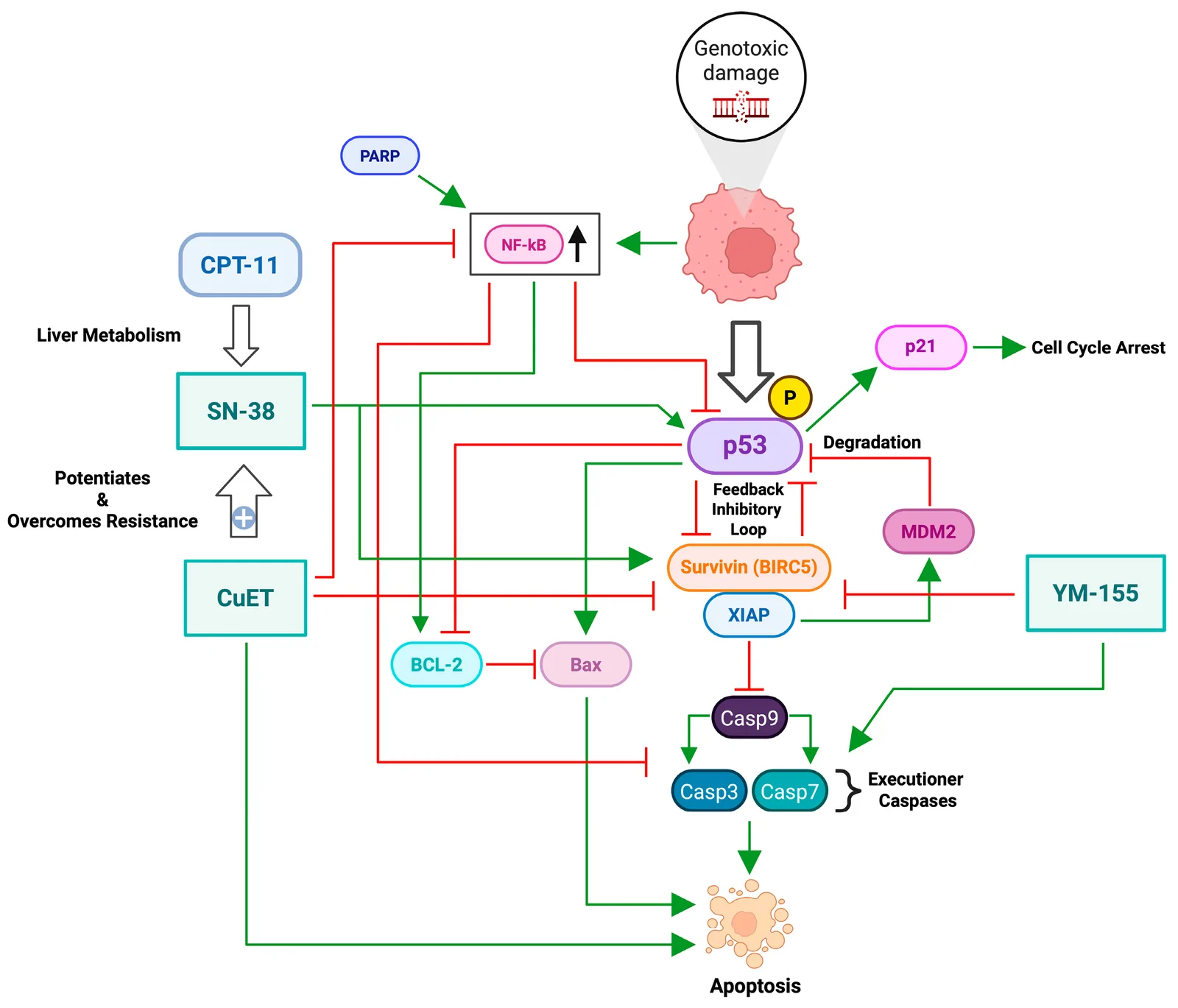
Mechanism of Survivin-targeted CRC sensitization to irinotecan by CuET and YM-155. CPT-11 (irinotecan), via its active metabolite SN-38, induces genotoxic stress and activates DNA damage response pathways, including p53 signaling, leading to p21-mediated cell cycle arrest and activation of intrinsic apoptosis through Bax and caspase cascades. In *TP53*-mutant CRC, loss of functional p53 disrupts this apoptotic program and promotes compensatory activation of NF-κB-driven survival pathways, resulting in upregulation of anti-apoptotic mediators including Survivin (*BIRC5*), XIAP, and BCL-2, which collectively inhibit caspase-9 and executioner caspases (caspase-3/7), thereby conferring chemoresistance. Here, we integrate two complementary therapeutic strategies to overcome this resistance. Direct Survivin suppression using YM-155 abrogates SN-38–induced Survivin upregulation, restoring caspase-dependent apoptosis and enhancing cytotoxic efficacy. In parallel, the disulfiram metabolite CuET exerts p53-independent anticancer activity through partial suppression of Survivin expression in addition to its proteotoxic and NF-κB-inhibitory effects. These interventions converge on reactivation of apoptotic signaling, characterized by increased PARP cleavage and robust activation of executioner caspases. When combined with irinotecan, these approaches synergistically dismantle the apoptosis-resistant phenotype of *TP53*-mutant CRC, restoring therapeutic sensitivity and promoting tumor cell death. This integrated model positions Survivin signaling as an actionable vulnerability and supports CuET-based combination strategies as a promising approach to overcome chemoresistance in CRC.

Taken together, our results support a mechanistic model in which irinotecan-induced DNA damage triggers compensatory Survivin upregulation that suppresses caspase activation and limits tumor cell death. Pharmacologic suppression of Survivin or induction of proteotoxic stress disrupts this survival program, enabling robust activation of the intrinsic apoptotic cascade (Figure 6). Importantly, while YM-155 directly inhibits Survivin expression, additional mechanisms likely contribute to the activity of CuET. Although CuET exerts context-dependent effects on Survivin expression, it may produce broader anticancer activity by disrupting protein homeostasis and inhibiting survival signaling pathways, thereby restoring treatment sensitivity even in the absence of functional p53.

Several limitations of this study warrant consideration. First, although SN-38 exposure was consistently associated with increased Survivin expression in both *TP53*-proficient and -deficient cells, the present experiments did not establish the upstream mechanism responsible for this response. Because *BIRC5* transcription, promoter activity, upstream signaling pathways, and Survivin protein stability were not directly assessed, Survivin induction should be interpreted as a treatment-associated adaptive response rather than evidence of a single causal resistance mechanism. In addition, although caspase-3/-7 and PARP cleavage together with Annexin V staining support engagement of apoptotic signaling, the present experiments do not establish caspase-dependent apoptosis as the exclusive mechanism underlying the enhanced cytotoxicity of the YM-155/SN-38 combination. The contribution of caspase-independent apoptosis and other regulated cell-death pathways therefore warrants further investigation. Second, although our findings were evaluated in multiple two-dimensional cell models, three-dimensional spheroids, and two *in vivo* models, additional validation in genetically diverse CRC models, patient-derived cells or organoids, and models of acquired irinotecan resistance will be required to establish their broader generalizability. Third, while CuET attenuated treatment-associated Survivin expression, the molecular basis of this effect remains unclear. Future studies should determine whether CuET regulates Survivin through proteotoxic stress, NF-κB signaling, altered *BIRC5* transcription, changes in protein stability, or a combination of these mechanisms. Finally, pharmacokinetic optimization and comprehensive toxicity studies will be necessary to determine whether CuET-based therapies can be safely integrated into existing chemotherapy regimens.

## 5. Conclusions

In summary, this study identifies Survivin-driven apoptotic resistance as a key determinant of irinotecan response in CRC and demonstrates that targeting this pathway restores tumor sensitivity to chemotherapy. Our findings further establish CuET as a potent p53-independent anticancer agent capable of overcoming irinotecan resistance in *TP53*-mutant CRC, supporting its potential as a therapeutic strategy for a substantial subset of patients with limited treatment options. Collectively, these results provide a strong rationale for the clinical evaluation of CuET-based combination therapies, with the potential to enhance responses to irinotecan-containing regimens and improve outcomes in chemoresistant CRC.

## Data Availability

The data presented in this study are available from the corresponding author upon reasonable request. The data are not publicly available due to their use in ongoing research projects.

## Supplementary Materials

The following supporting information can be downloaded at: https://www.mdpi.com/article/10.3390/cancers18162687/s1, Figure S1: Quantification of Survivin protein expression in cell lysates from HCT116 Parental and WT treated with SN-38 (10 to 50 nM) and/or YM-155 (1 to 15 nM) for 48 h; Figure S2: Representative brightfield images and cell count of HCT116 Parental cells treated with DMSO control, SN-38 (20 nM), YM-155 (20 nM), or SN-38 plus YM-155, for 72 h; Figure S3: Quantification of caspases 3 and 7, PARP, XIAP, BAX, and p21 protein expression in cell lysates from HCT116 Parental and WT treated with SN-38 (10 to 50 nM) and/or YM-155 (1 to 15 nM) for 48 h; Figure S4: Representative selection of whole-section TUNEL staining of HCT116 xenograft tumors following YM-155, CPT-11, or combination treatment (scale bar 1 mm); Figure S5: Evaluation of systemic toxicity in major internal organs from treated MC-38 tumor-bearing mice.

## Abbreviations

The following abbreviations are used in this manuscript:

*APC*	Adenomatous Polyposis Coli
ATCC	American Type Culture Collection
BAX	Pro-Apoptotic Bcl-2-Associated X Protein
BCA	Bicinchoninic Acid Assay
BCL-2	B-Cell Lymphoma 2
*BIRC-5*	Baculoviral Iap Repeat Containing 5
*BRAF*	B-Raf Proto-Oncogene, Serine/Threonine Kinase
CI	Combination Index
CuET	Bis(Diethyldithiocarbamato)Copper
CPT-11	Irinotecan
CRC	Colorectal Cancer
DAB	3,3′-Diaminobenzidine
DMEM	Dulbecco’s Modified Eagle Medium
DMSO	Dimethyl Sulfoxide
DNA	Deoxyribonucleic Acid
DSF	Disulfiram
ECL	Enhanced Chemiluminescence
EDTA	Ethylenediaminetetraacetic Acid
FACC	Facility Animal Care Committee
FBS	Fetal Bovine Serum
FITC	Fluorescein Isothiocyanate
FOLFIRI	Folinic Acid, 5-Fluorouracil, Irinotecan
FOLFOX	Folinic Acid, Fluorouracil, Oxaliplatin
HRP	Horseradish Peroxidase
IAP	Inhibitor-Of-Apoptosis
IC50	Inhibitory Concentration 50
IHC	Immunohistochemistry
*KRAS*	Kirsten Rat Sarcoma Viral Oncogene Homolog
MSA	Mouse Serum Albumin
NF-κB	Nuclear Factor Kappa-Light-Chain-Enhancer Of Activated B Cells
OD	Optical Density
PARP	Poly ADP-Ribose Polymerase
PBS	Phosphate-Buffered Saline
PVDF	Polyvinylidene Fluoride
RIPA	Radioimmunoprecipitation Assay Buffer
RNA	Ribonucleic Acid
SCID	Severe Combined Immunodeficiency
SD	Standard Deviation
SDS	Sodium Dodecyl Sulfate
SDS-PAGE	Sodium Dodecyl Sulfate–Polyacrylamide Gel Electrophoresis
SEM	Standard Error To The Mean
SN-38	7-Ethyl-10-Hydroxycamptothecin; Irinotecan Active Metabolite
SRB	Sulforhodamine B
*TP-53*	Tumor Protein P53
*Trp-53*	Transformation-Related Protein 53
TUNEL	Terminal Deoxynucleotidyl Transferase (Tdt) Dutp Nick End Labeling
WT	Wild-Type
XIAP	X-Linked Inhibitor Of Apoptosis Protein
XTT	2,3-Bis-(2-Methoxy-4-Nitro-5-Sulfophenyl)-2H-Tetrazolium-5-Carboxanilide assay
YM-155	Sepantronium Bromide
