# Supplementary material for: Overcoming Resistance: Targeting Survivin-Driven Apoptotic Resistance Restores Irinotecan Sensitivity in TP53-Mutant Colorectal Cancer
Source: Cancers (Basel). 2026 Aug 19;18(16):2687. doi: 10.3390/cancers18162687 (PMC13511531; doi:10.3390/cancers18162687)
Supplement: Supplementary file 1 [file cancers-18-02687-s001.zip › cancers-4323027-supplementary.pdf]

Supplementary Materials

Overcoming Resistance: Targeting Survivin-Driven Apoptotic Resistance Restores Irinotecan Sensitivity in *TP53*-Mutant Colorectal Cancer

Daciana Catalina Dumut [1,2], Yong Zhong Xu [1,2,†], Daniela Verelli [2,3,†], Viswanath Das [4,5], Marian Hajduch [4,5], Juan Bautista DeSanctis [4,5] and Danuta Radzioch [1,2,4,*]

1 Department of Experimental Medicine, Faculty of Medicine, McGill University, Montreal, QC H3A 1Y2, Canada; daciana.dumut@mail.mcgill.ca (D.C.D.); yong.xu@mail.mcgill.ca (Y.Z.X.)
2 Infectious Diseases in Global Health Program, The Research Institute of the McGill University Health Centre, Montreal, QC H3A 1A1, Canada; daniela.verelli@mail.mcgill.ca
3 Department of Microbiology and Immunology, Faculty of Medicine, McGill University, Montreal, QC H3A 0G4, Canada
4 Institute of Molecular and Translational Medicine, Faculty of Medicine and Dentistry, Palacky University, 77900 Olomouc, Czech Republic; viswanath.das@upol.cz (V.D.); marian.hajduch@upol.cz (M.H.); juanbautista.desanctis@upol.cz (J.B.D.)
5 Czech Advanced Technology and Research Institute, Palacky University Olomouc, Krizkovskeho 511/8, 77900 Olomouc, Czech Republic
* Correspondence: danuta.radzioch@mcgill.ca
† These authors contributed equally to this work.

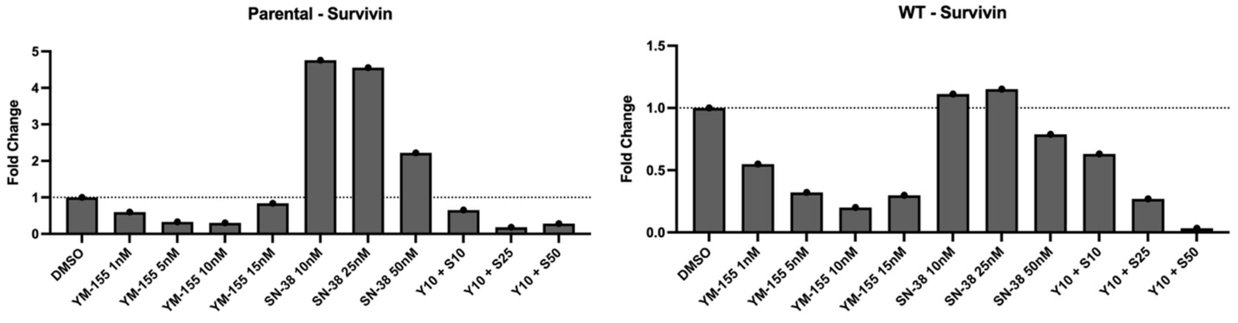

Figure S1. Quantification of Survivin protein expression in cell lysates from HCT116 Parental and WT treated with SN-38 (10 to 50nM) and/or YM-155 (1 to 15nM) for 48h.

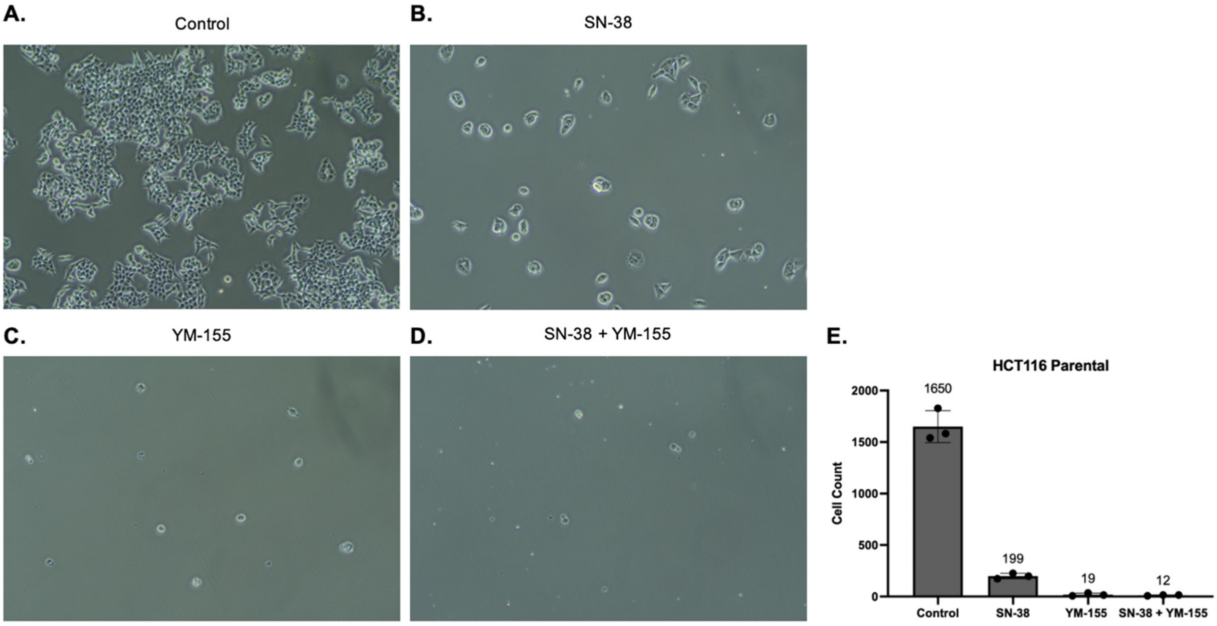

Figure S2. Representative brightfield images and cell count of HCT116 Parental cells treated with DMSO control, SN-38 (20 nM), YM-155 (20 nM), or SN-38 plus YM-155, for 72h. Cell count allows appreciation of cytotoxic-associated loss of adherent cells before immunofluorescence staining (Shown in manuscript Figure 2D,F).

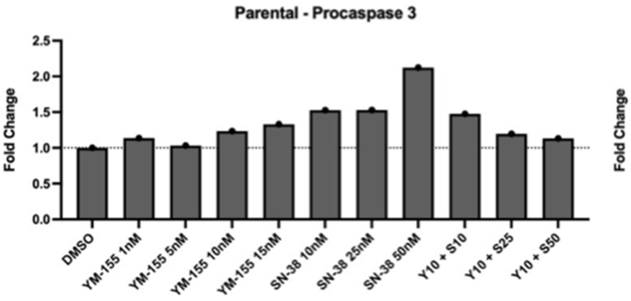
Parental - Procaspase 3
Fold Change
2.5
2.0
1.5
1.0
0.5
0.0
DMSO
YM-155 1nM
YM-155 5nM
YM-155 10nM
YM-155 15nM
SN-38 10nM
SN-38 25nM
SN-38 50nM
Y10 + S10
Y10 + S25
Y10 + S50

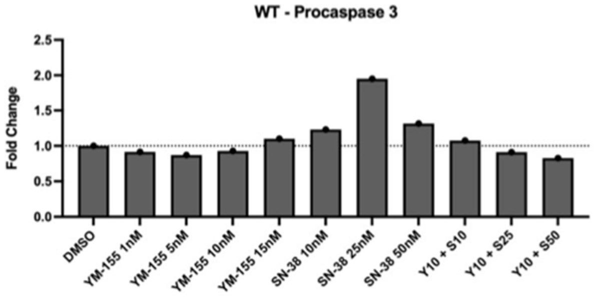
WT - Procaspase 3
Fold Change
2.5
2.0
1.5
1.0
0.5
0.0
DMSO
YM-155 1nM
YM-155 5nM
YM-155 10nM
YM-155 15nM
SN-38 10nM
SN-38 25nM
SN-38 50nM
Y10 + S10
Y10 + S25
Y10 + S50

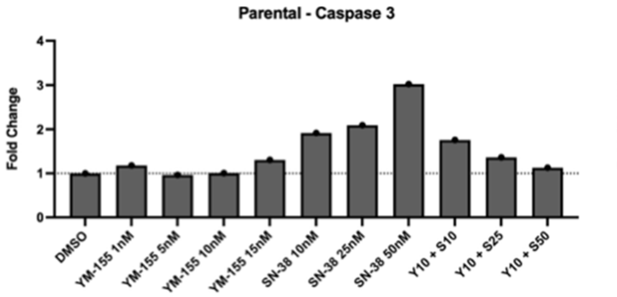
Parental - Caspase 3
Fold Change
4
3
2
1
0
DMSO
YM-155 1nM
YM-155 5nM
YM-155 10nM
YM-155 15nM
SN-38 10nM
SN-38 25nM
SN-38 50nM
Y10 + S10
Y10 + S25
Y10 + S50

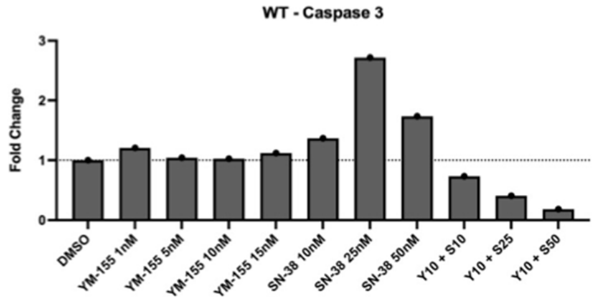
WT - Caspase 3
Fold Change
3
2
1
0
DMSO
YM-155 1nM
YM-155 5nM
YM-155 10nM
YM-155 15nM
SN-38 10nM
SN-38 25nM
SN-38 50nM
Y10 + S10
Y10 + S25
Y10 + S50

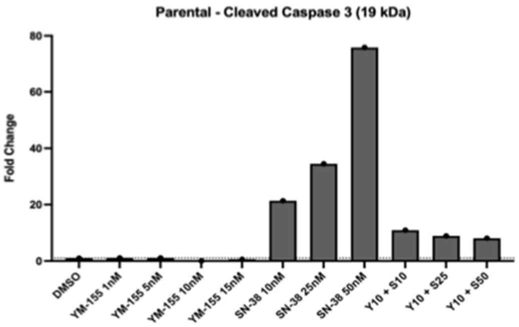
Parental - Cleaved Caspase 3 (19 kDa)
Fold Change
80
60
40
20
0
DMSO
YM-155 1nM
YM-155 5nM
YM-155 10nM
YM-155 15nM
SN-38 10nM
SN-38 25nM
SN-38 50nM
Y10 + S10
Y10 + S25
Y10 + S50

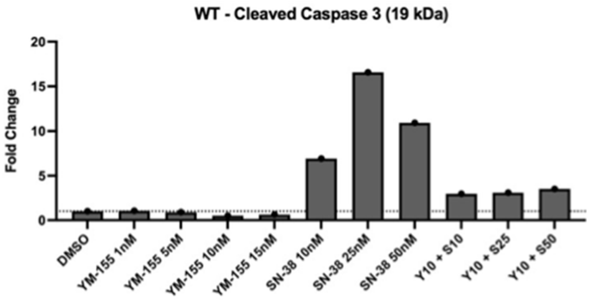
WT - Cleaved Caspase 3 (19 kDa)
Fold Change
20
15
10
5
0
DMSO
YM-155 1nM
YM-155 5nM
YM-155 10nM
YM-155 15nM
SN-38 10nM
SN-38 25nM
SN-38 50nM
Y10 + S10
Y10 + S25
Y10 + S50

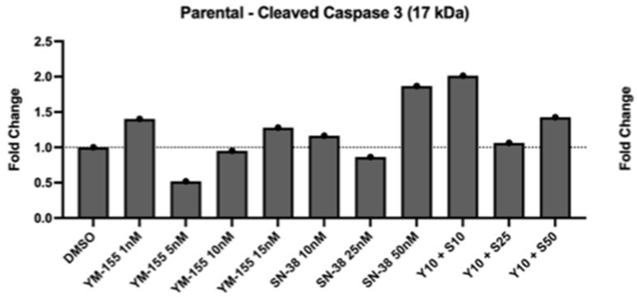
Parental - Cleaved Caspase 3 (17 kDa)
Fold Change
2.5
2.0
1.5
1.0
0.5
0.0
DMSO
YM-155 1nM
YM-155 5nM
YM-155 10nM
YM-155 15nM
SN-38 10nM
SN-38 25nM
SN-38 50nM
Y10 + S10
Y10 + S25
Y10 + S50

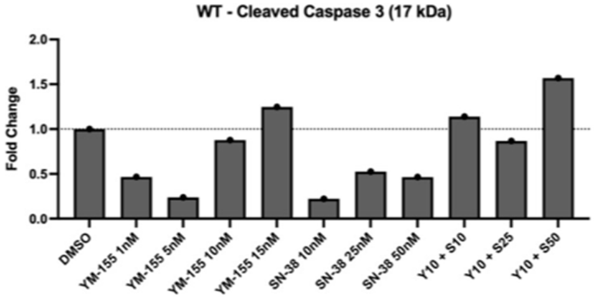
WT - Cleaved Caspase 3 (17 kDa)
Fold Change
2.0
1.5
1.0
0.5
0.0
DMSO
YM-155 1nM
YM-155 5nM
YM-155 10nM
YM-155 15nM
SN-38 10nM
SN-38 25nM
SN-38 50nM
Y10 + S10
Y10 + S25
Y10 + S50

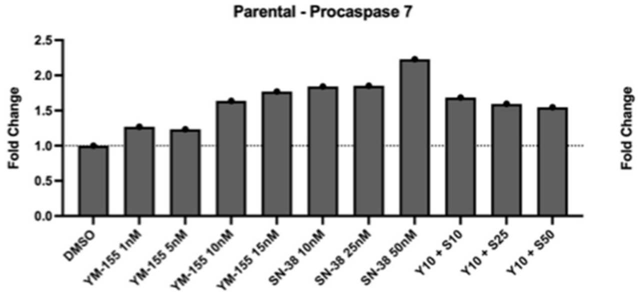
Parental - Procaspase 7
Fold Change
2.5
2.0
1.5
1.0
0.5
0.0
DMSO
YM-155 1nM
YM-155 5nM
YM-155 10nM
YM-155 15nM
SN-38 10nM
SN-38 25nM
SN-38 50nM
Y10 + S10
Y10 + S25
Y10 + S50

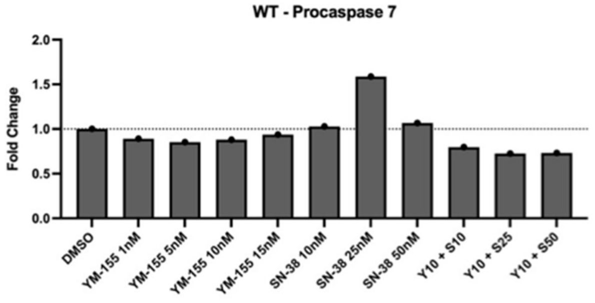
WT - Procaspase 7
Fold Change
2.0
1.5
1.0
0.5
0.0
DMSO
YM-155 1nM
YM-155 5nM
YM-155 10nM
YM-155 15nM
SN-38 10nM
SN-38 25nM
SN-38 50nM
Y10 + S10
Y10 + S25
Y10 + S50

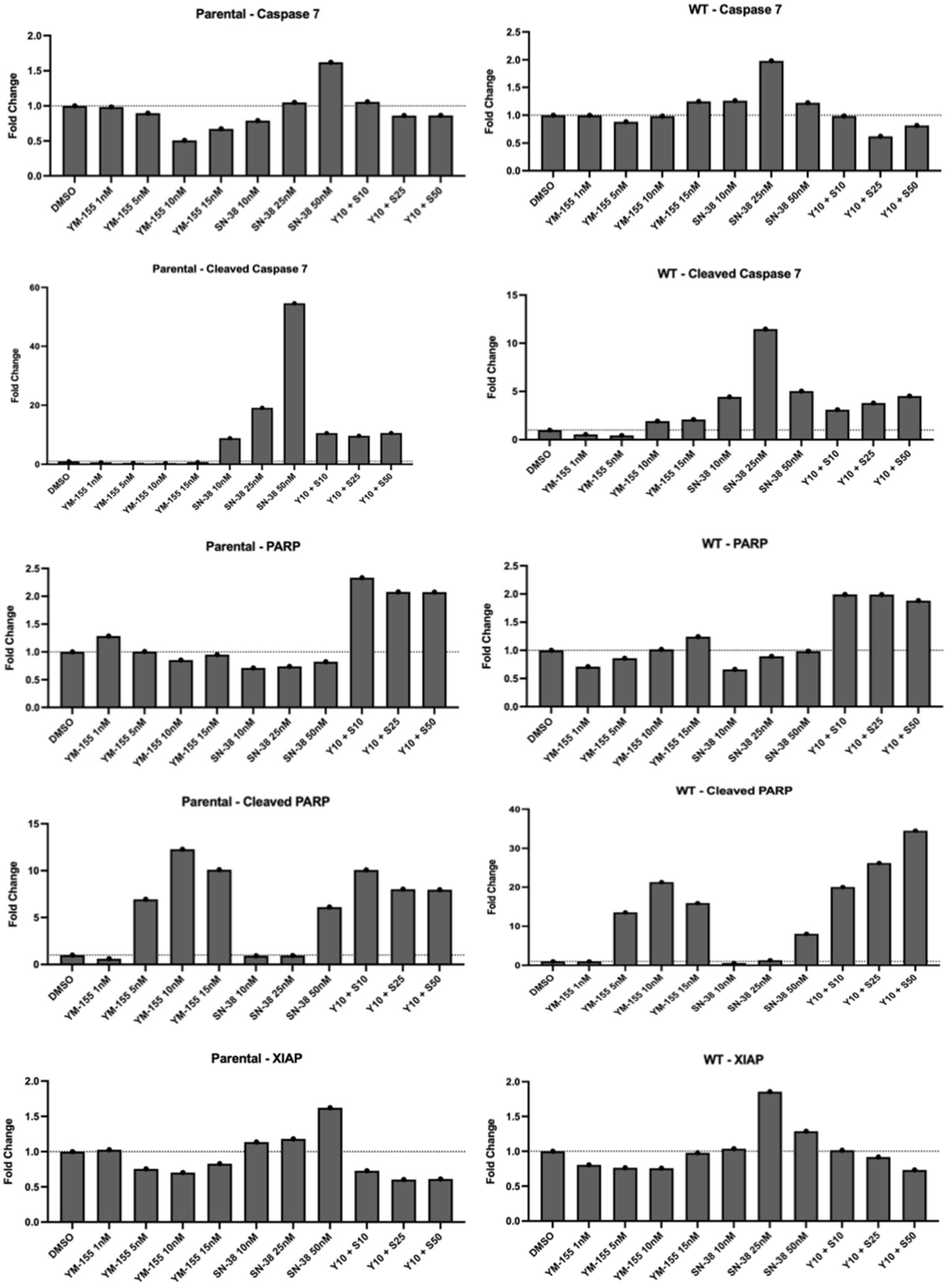
Parental - Caspase 7
WT - Caspase 7
Parental - Cleaved Caspase 7
WT - Cleaved Caspase 7
Parental - PARP
WT - PARP
Parental - Cleaved PARP
WT - Cleaved PARP
Parental - XIAP
WT - XIAP
Fold Change
DMSO
YM-155 1nM
YM-155 5nM
YM-155 10nM
YM-155 15nM
SN-38 10nM
SN-38 25nM
SN-38 50nM
Y10 + S10
Y10 + S25
Y10 + S50

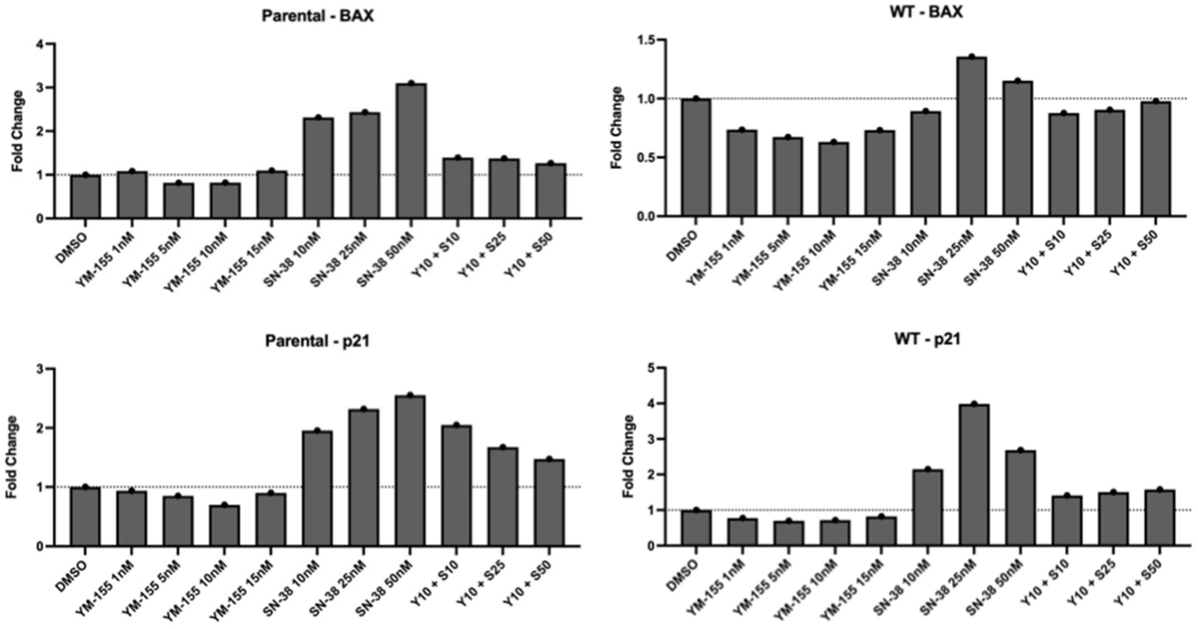

Figure S3. Quantification of caspases 3 and 7, PARP, XIAP, BAX, and p21 protein expression in cell lysates from HCT116 Parental and WT treated with SN-38 (10 to 50nM) and/or YM-155 (1 to 15nM) for 48h.

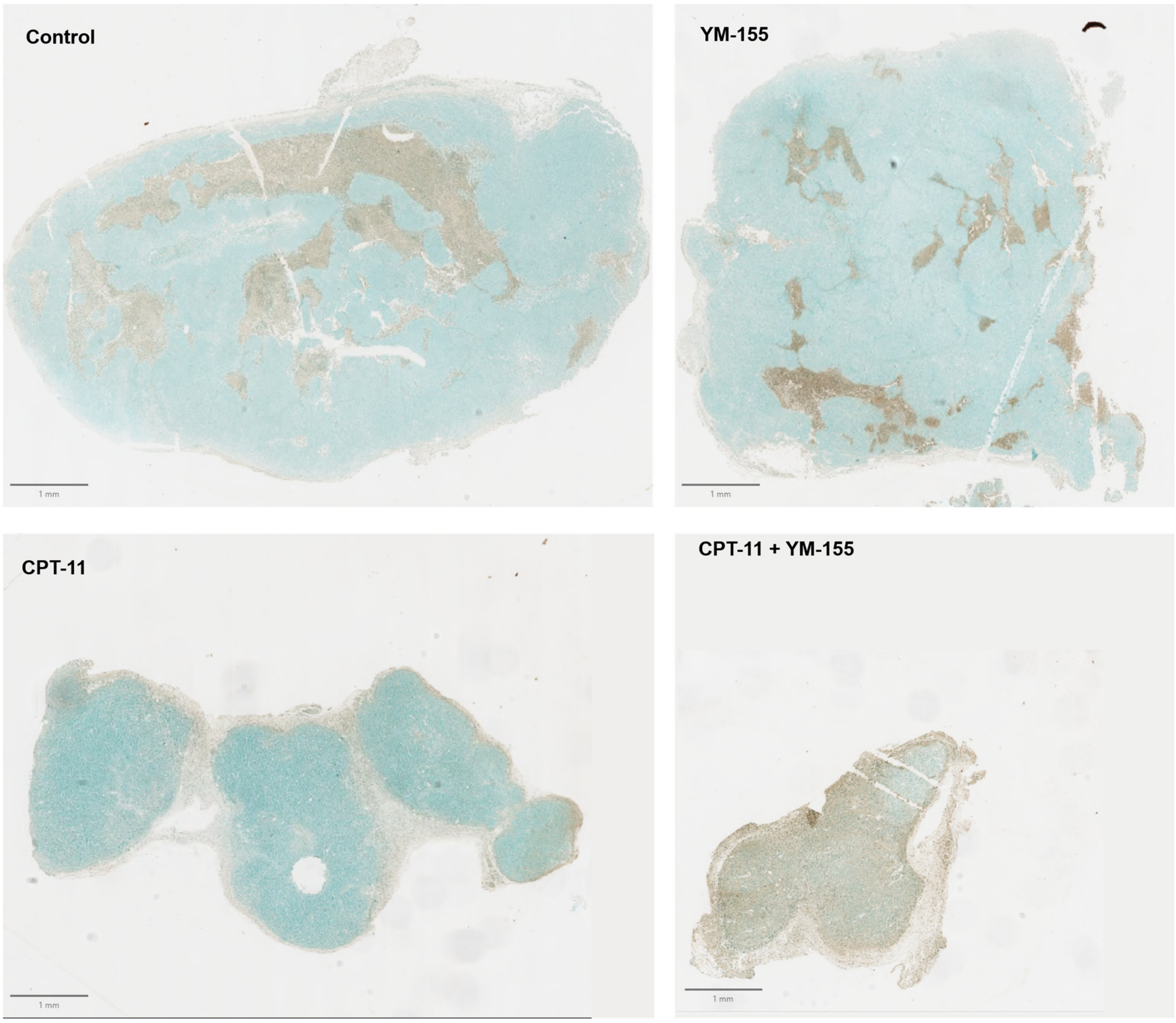

Figure S4. Representative selection of whole-section TUNEL staining of HCT116 xenograft tumors following YM-155, CPT-11, or combination treatment (scale bar 1mm).

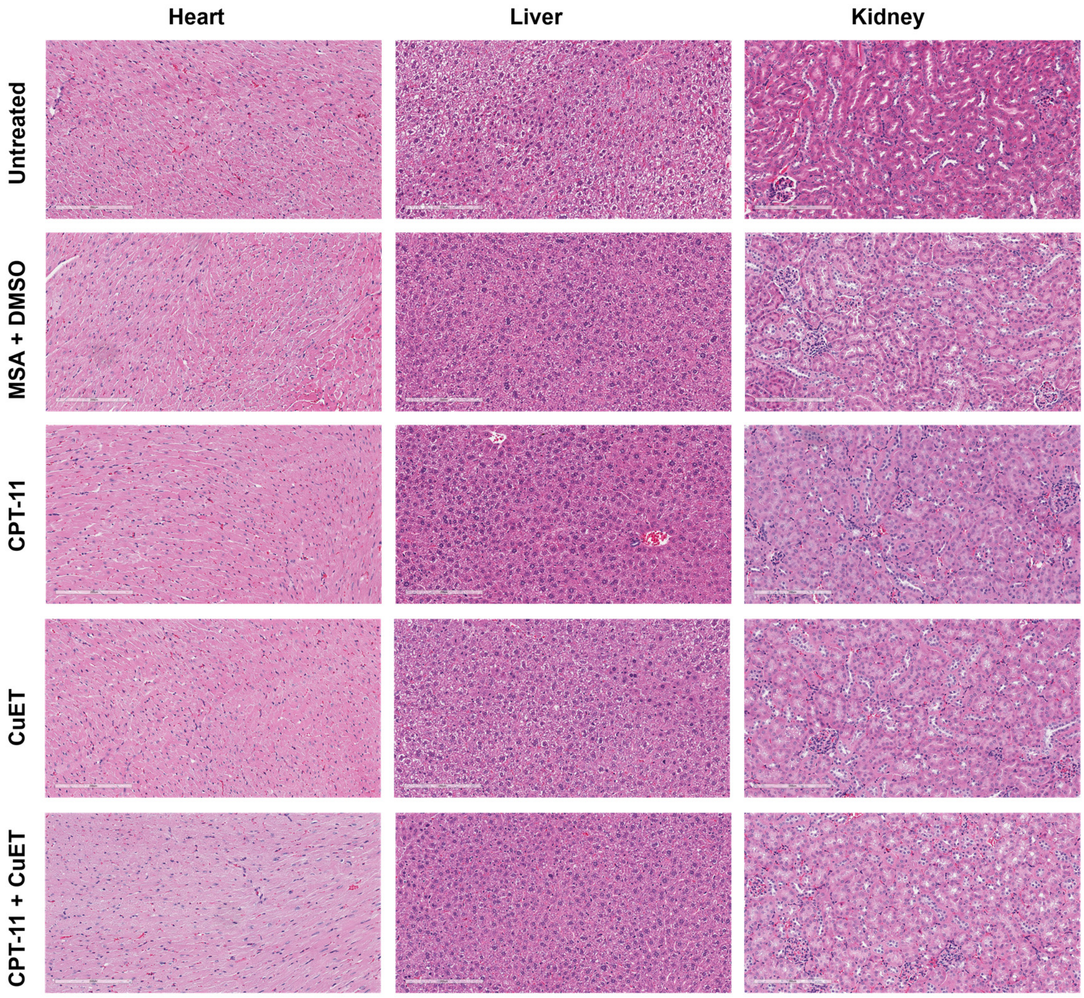

Figure S5. Evaluation of systemic toxicity in major internal organs from treated MC-38 tumor-bearing mice. Representative H&E-stained sections of heart, liver, and kidney from MC-38 colorectal tumor-bearing C57BL/6 mice treated with vehicle control (MSA + DMSO), CPT-11, CuET, or CPT-11 + CuET, alongside untreated controls. No overt treatment-associated histological alterations were observed in the examined organs, with overall preservation of cardiac, hepatic, and renal tissue morphology across groups. These data indicate that CuET, alone or combined with CPT-11, did not induce apparent organ toxicity under the conditions of this study. 20X magnification, 200μm scale-bars.
