# Supplementary material for: Overcoming Resistance: Targeting Survivin-Driven Apoptotic Resistance Restores Irinotecan Sensitivity in TP53-Mutant Colorectal Cancer
Source: Cancers (Basel). 2026 Aug 19;18(16):2687. doi: 10.3390/cancers18162687 (PMC13511531; doi:10.3390/cancers18162687)
Supplement: Supplementary file 1 [file cancers-18-02687-s001.zip › Survivin Manuscript - Original Blots.pdf]

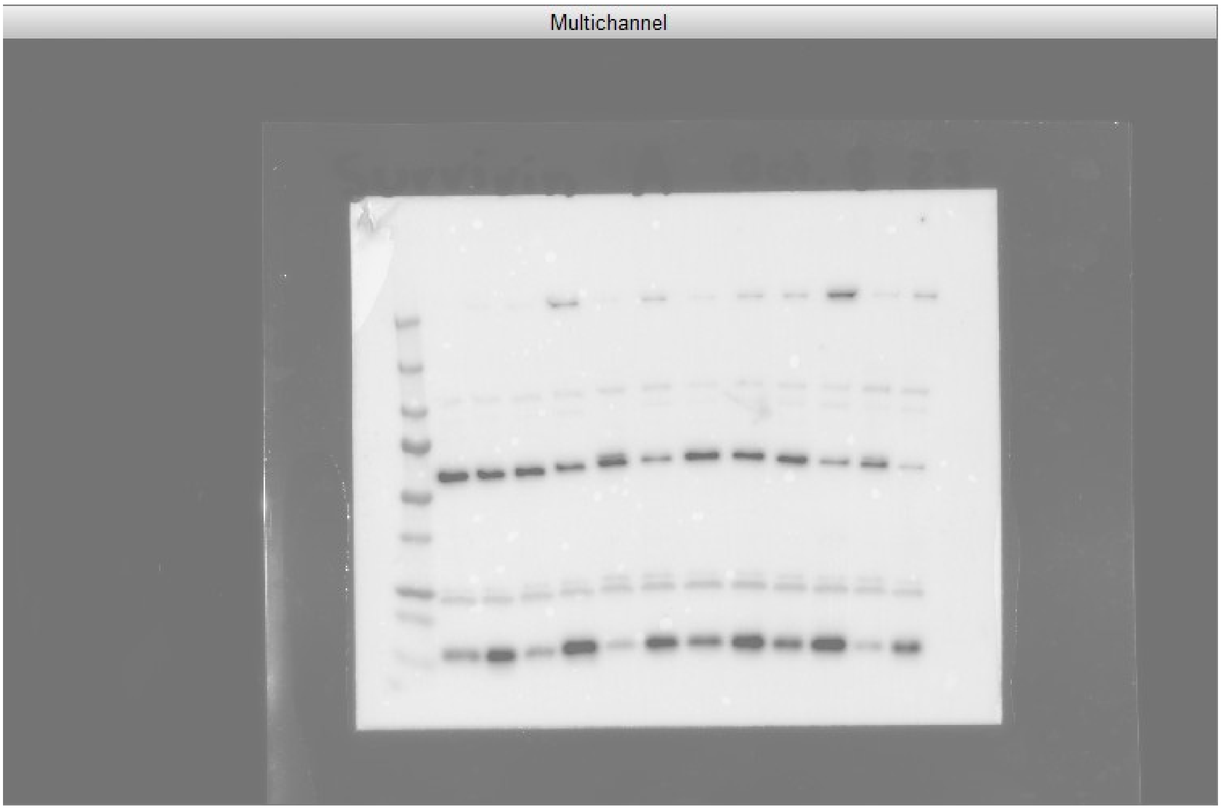

Image S1. Figure 2A. Survivin

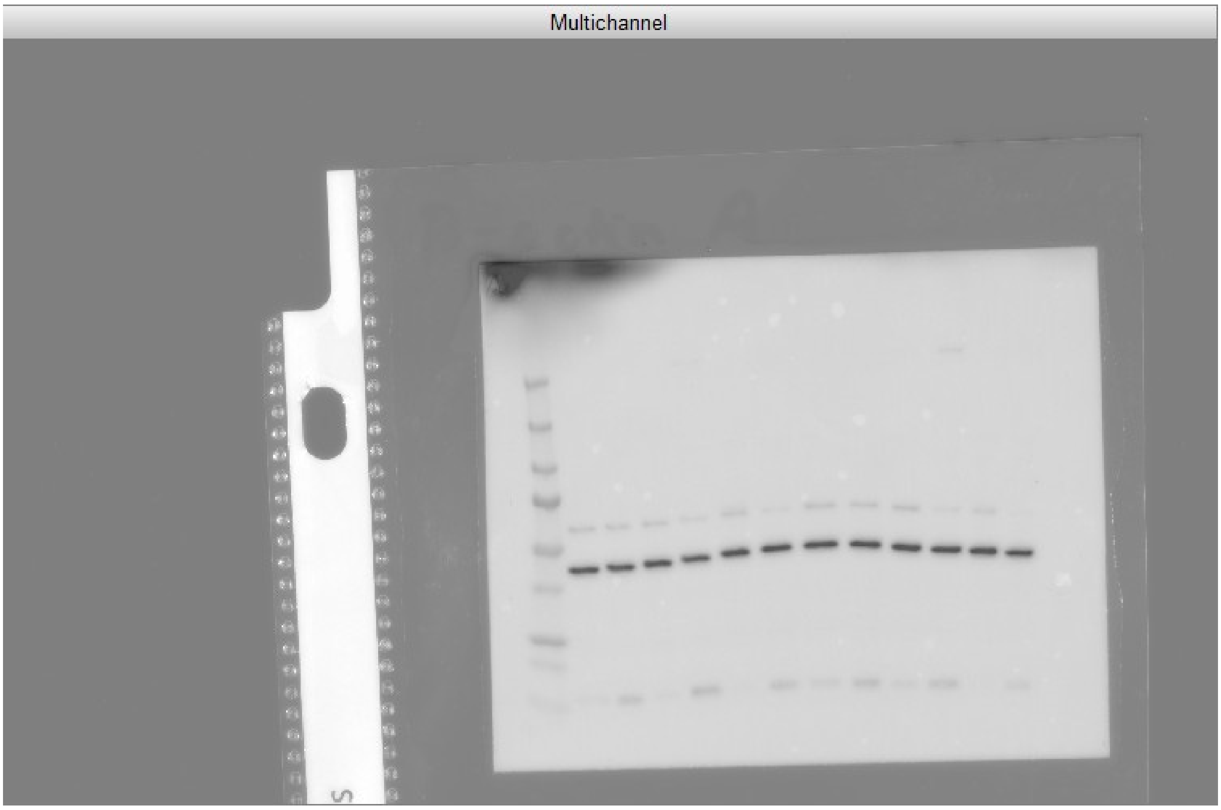

Image S2. Figure 2A. B-actin

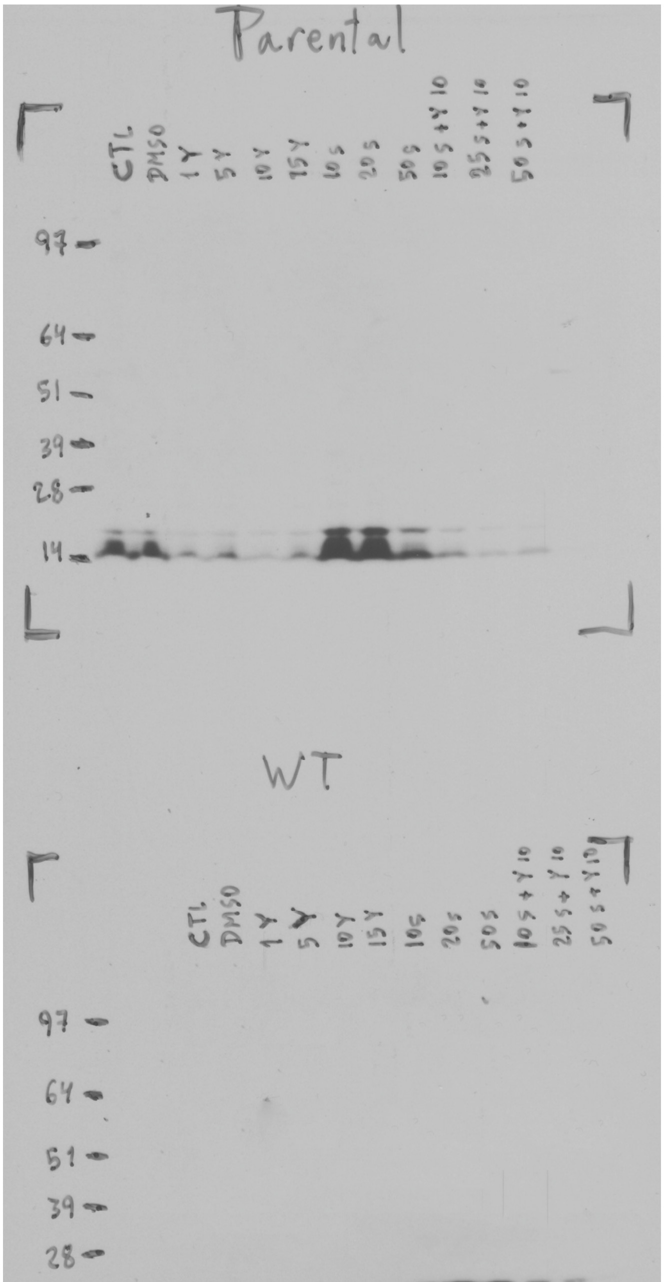

Image S3. Figure 2C. Survivin

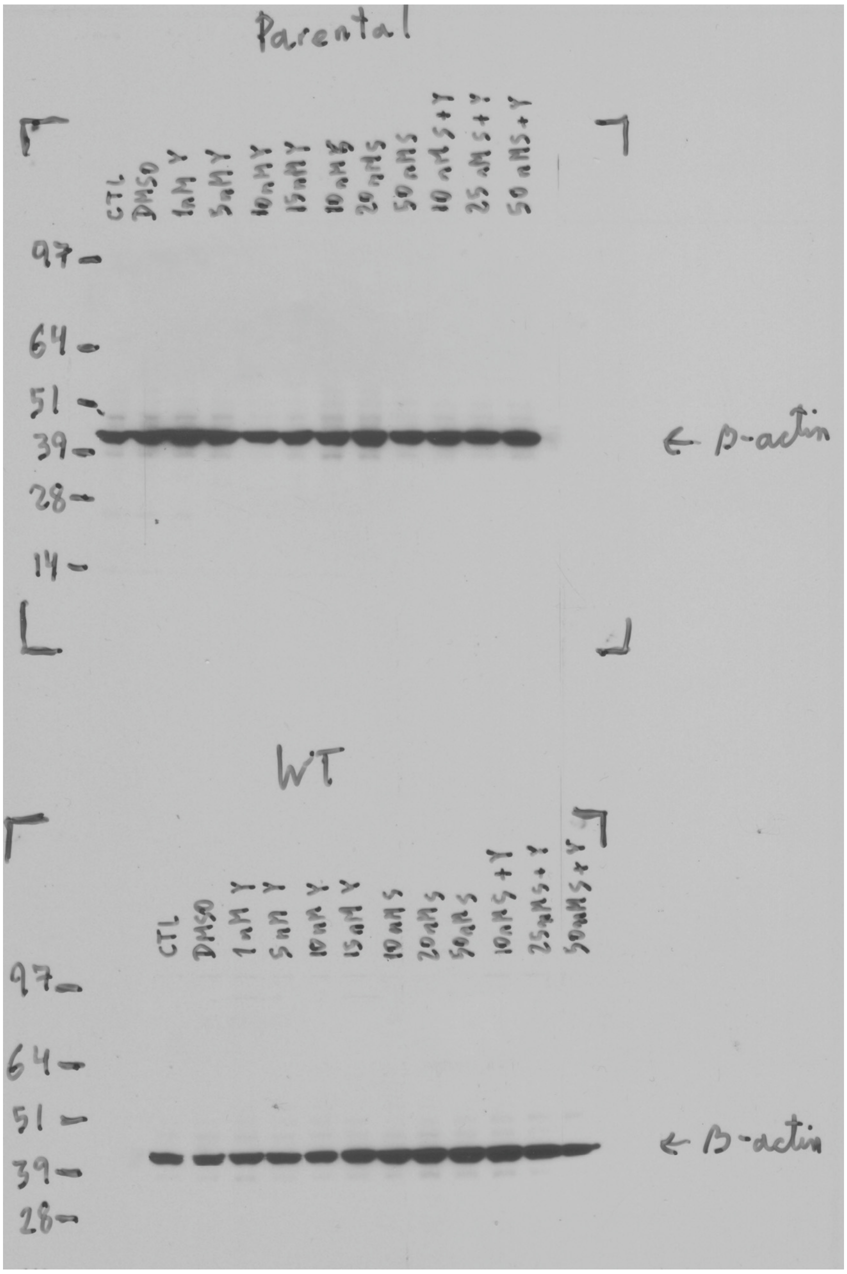

Image S4. Figure 2C. B-actin

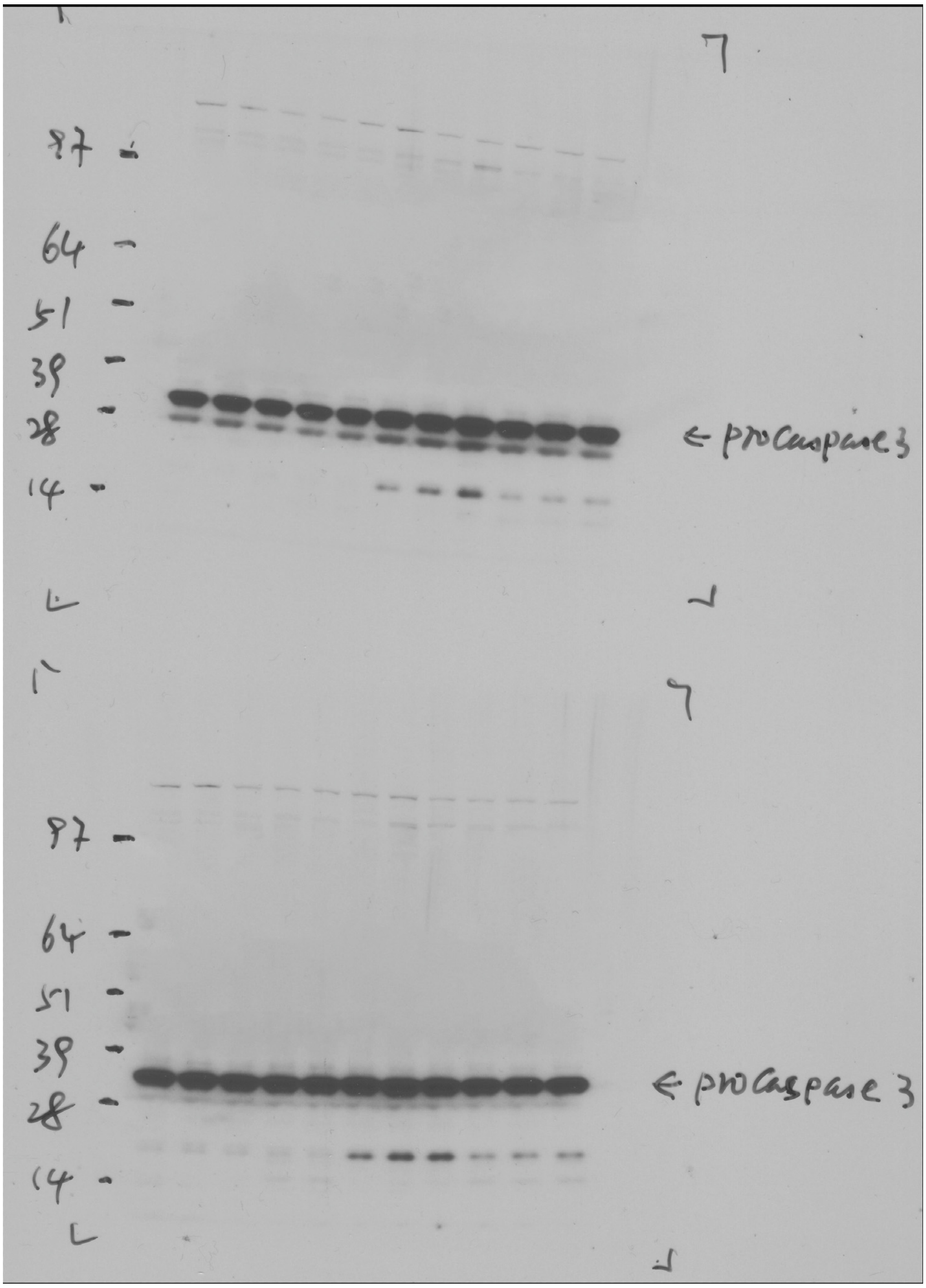

Image S5. Figure 2E. Procaspase 3, Caspase 3, Cleaved Caspase 3

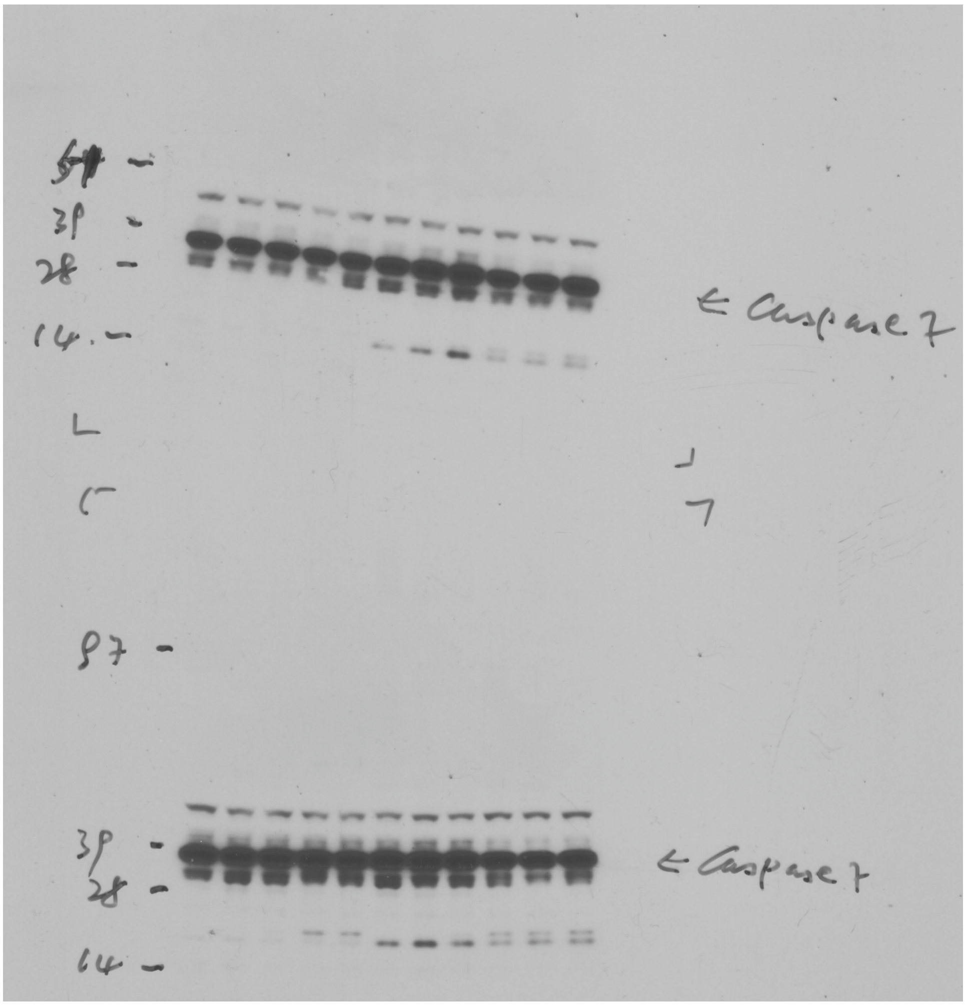

Image S6. Figure 2E. Procaspase 7, Caspase 7, Cleaved Caspase 7

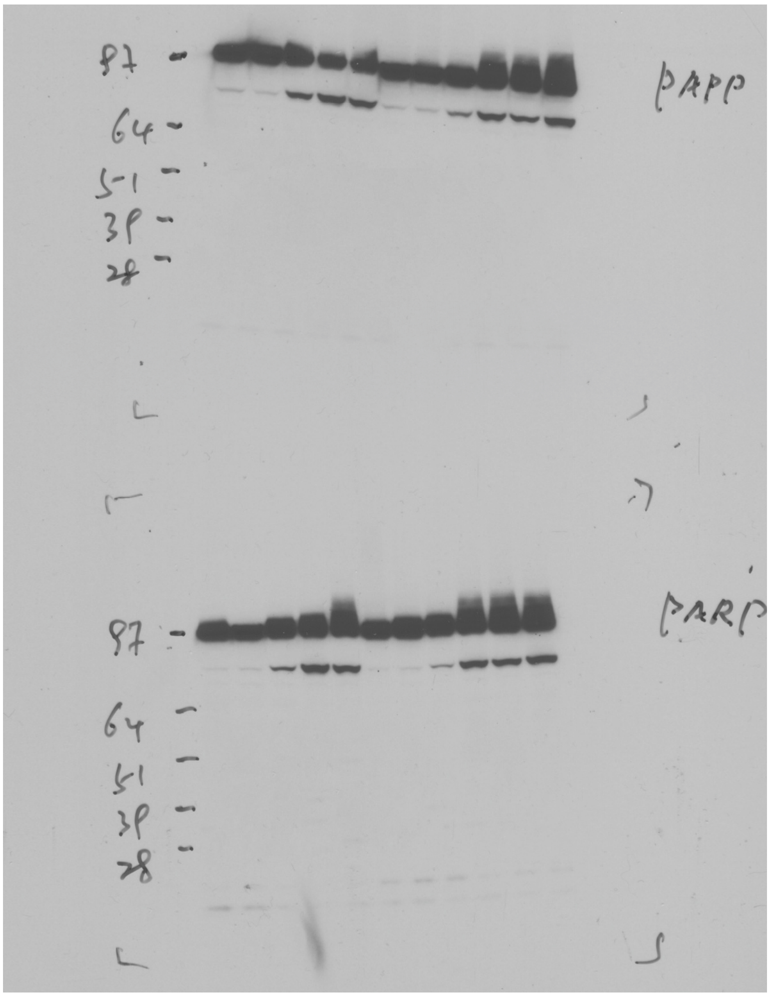

Image S7. Figure 2E. PARP, Cleaved PARP

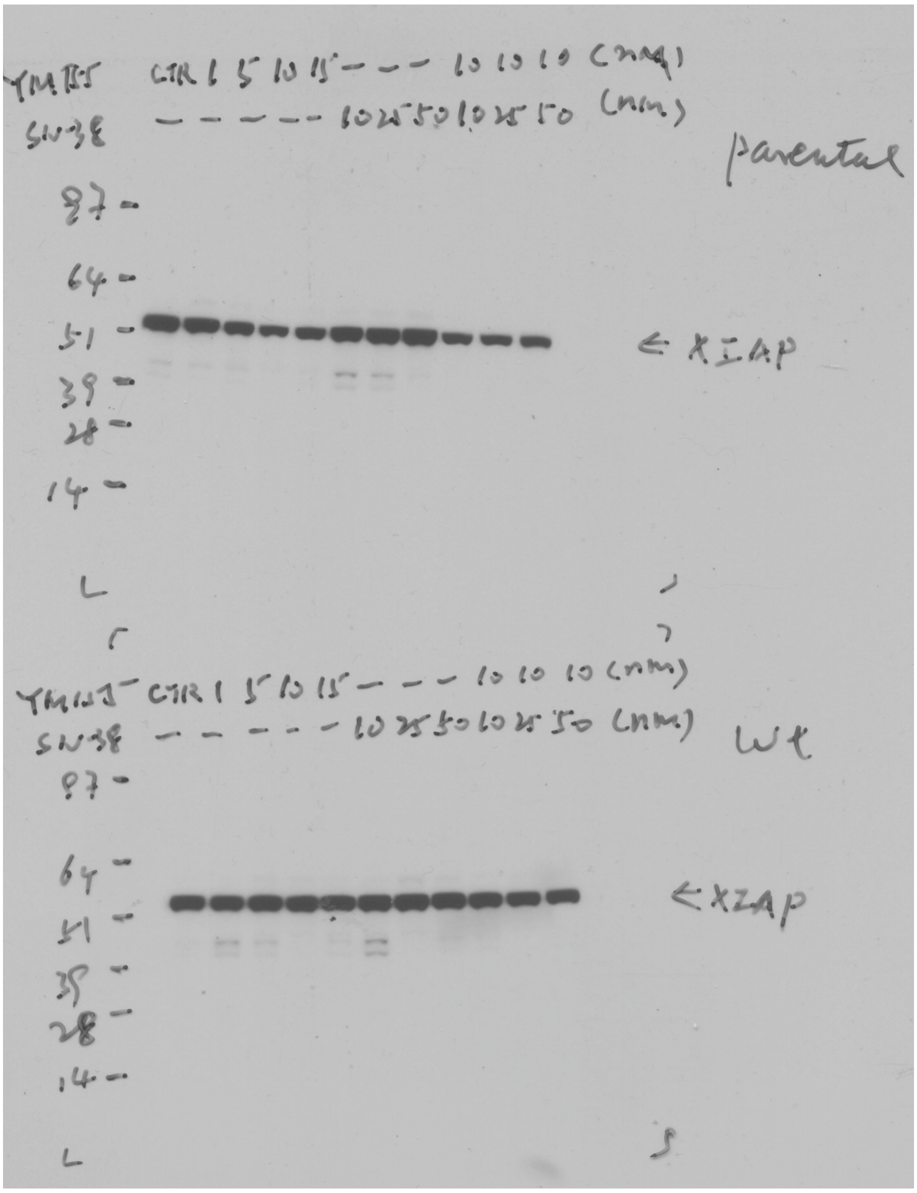

Image S8. Figure 2E. XIAP

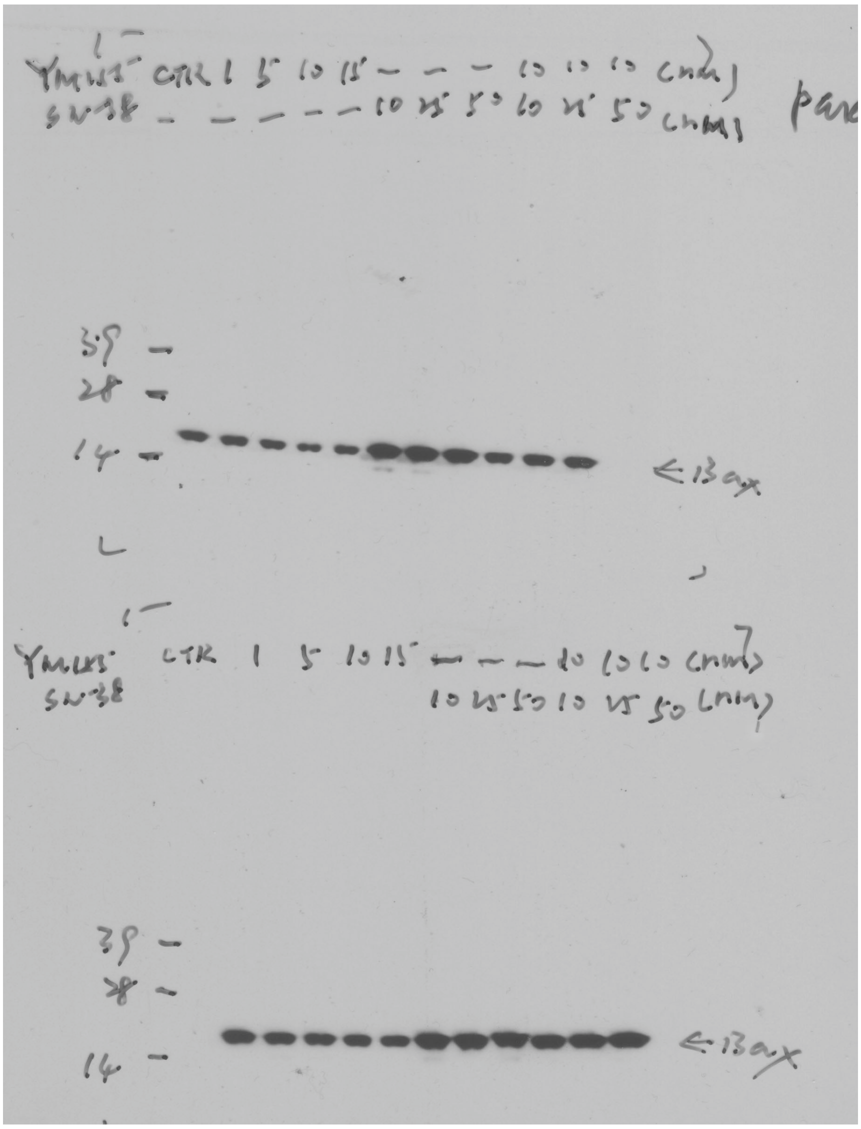

Image S9. Figure 2E. BAX

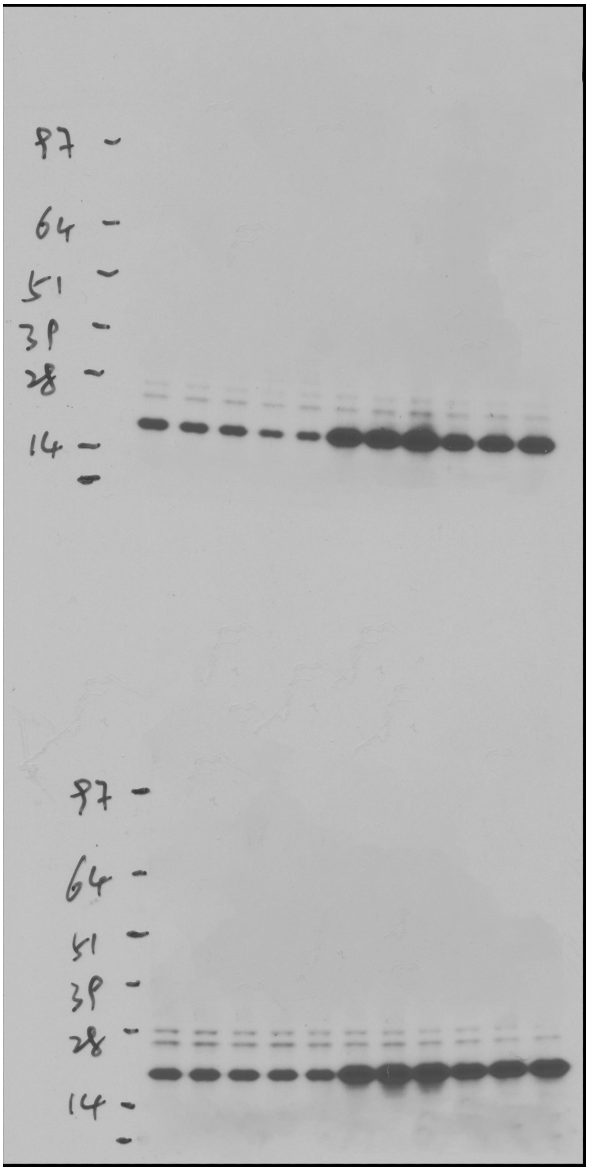

Image S10. Figure 2E. p21

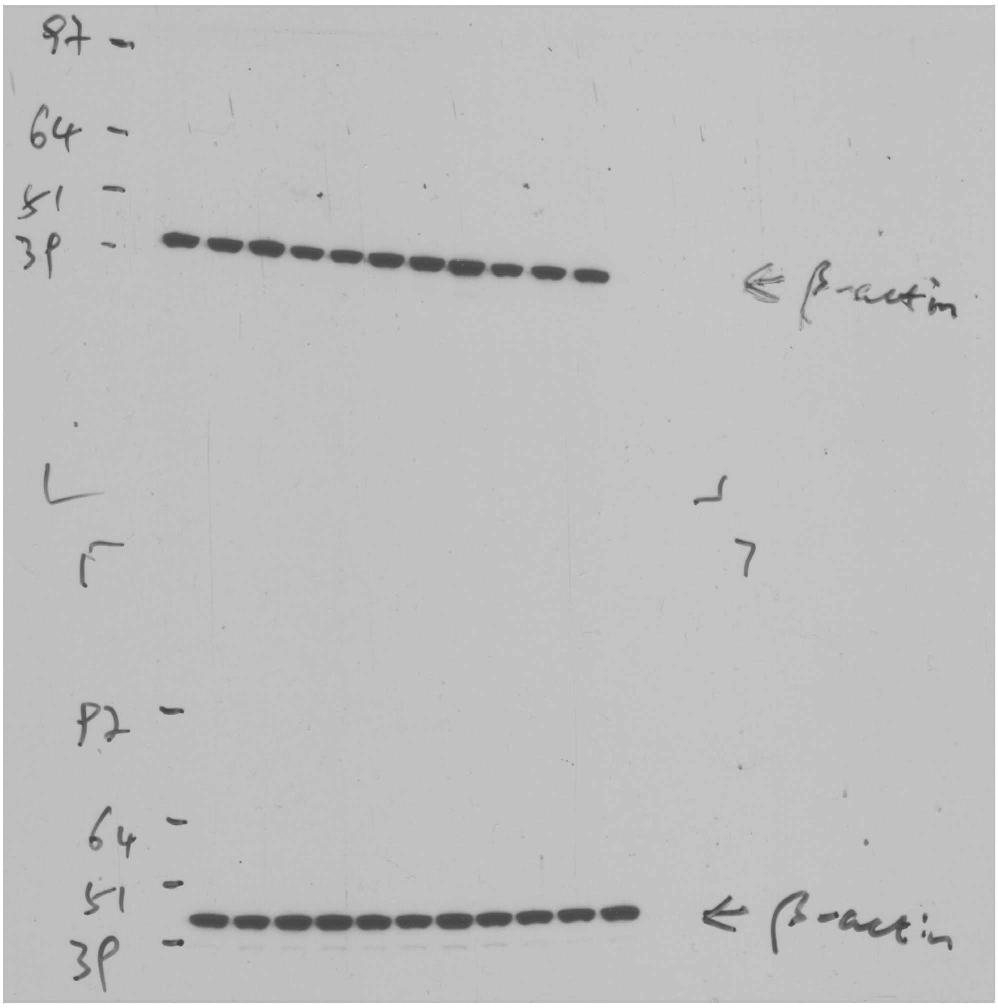

Image S11. Figure 2E. B-actin

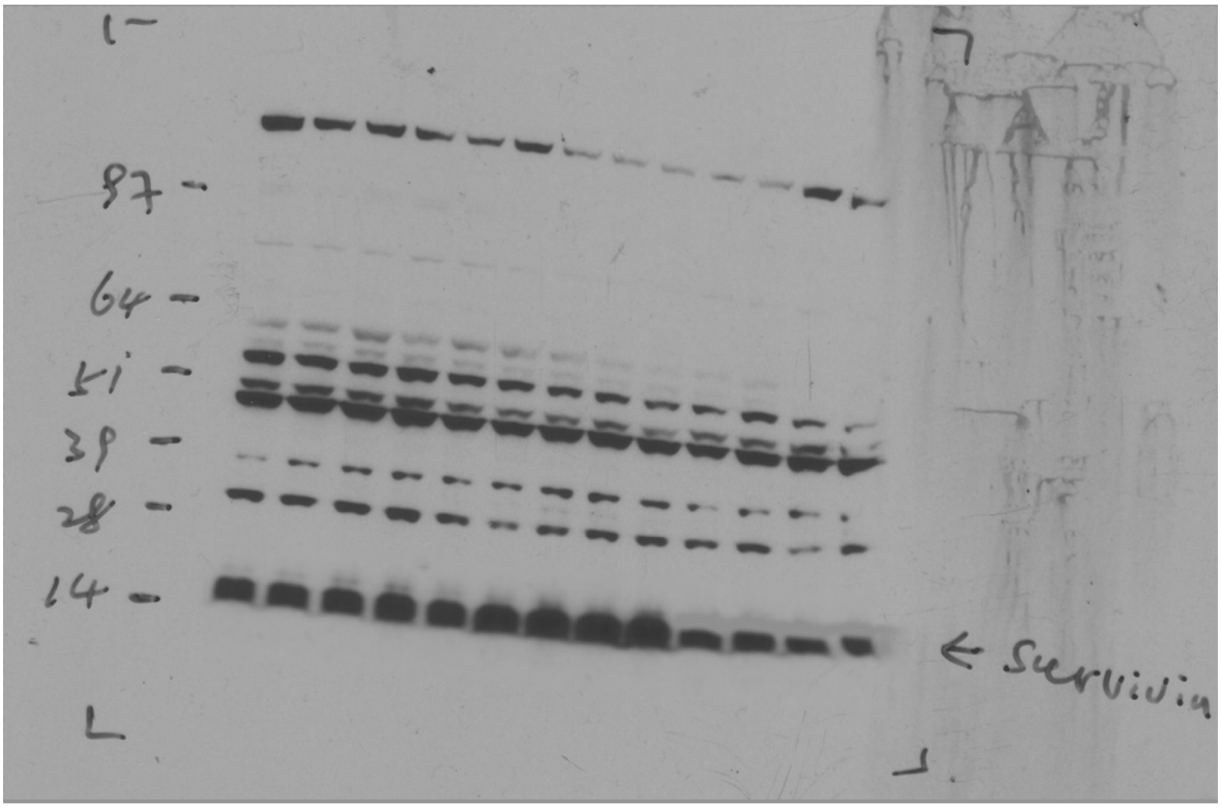

Image S12. Figure 3F. Survivin

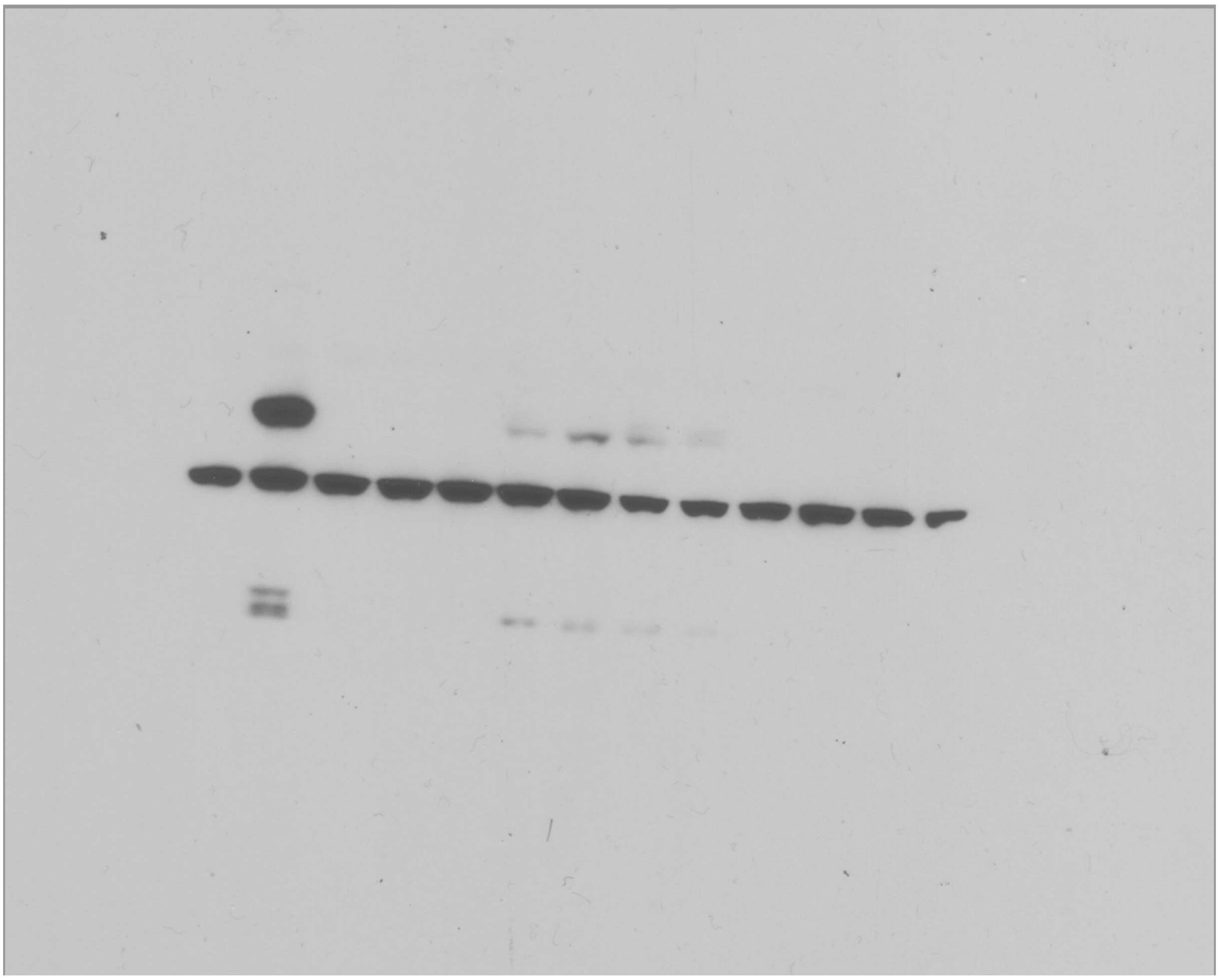

Image S13. Figure 3F. B-actin

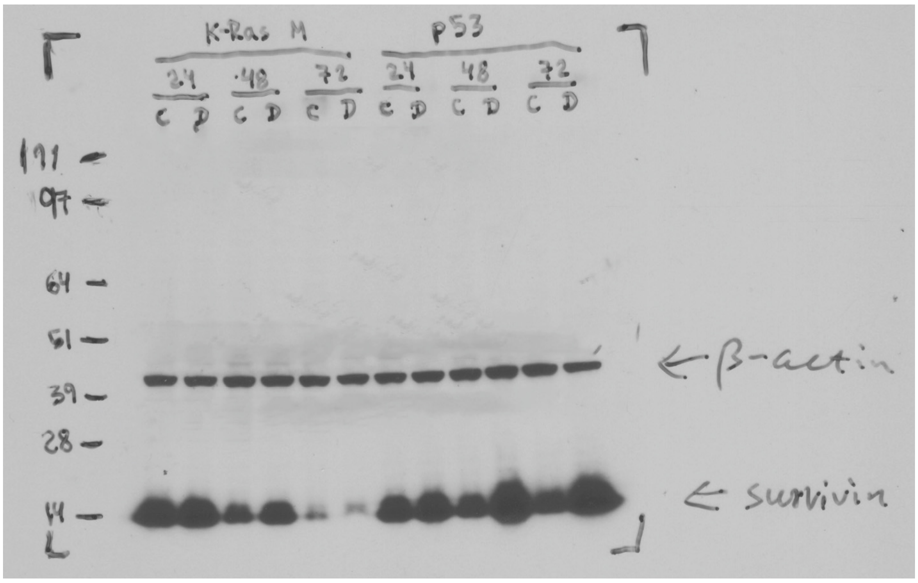

Image S14. Figure 4E. B-actin, Survivin

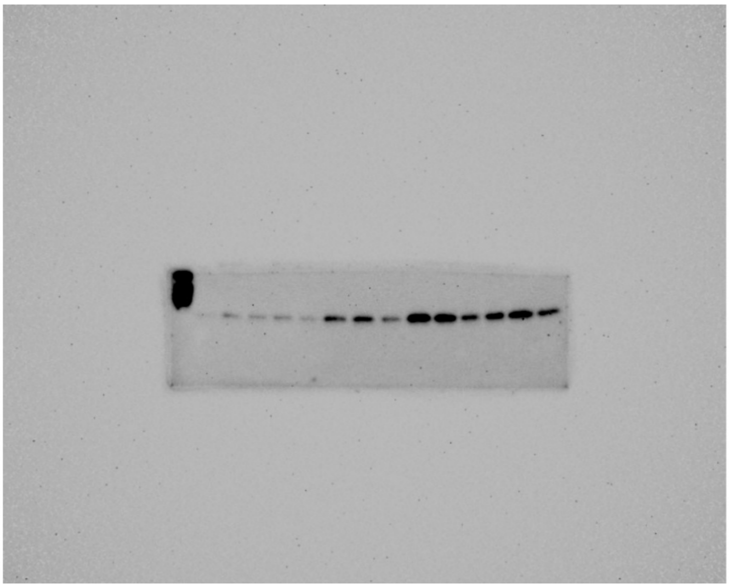

Image S15. Figure 4F. Survivin

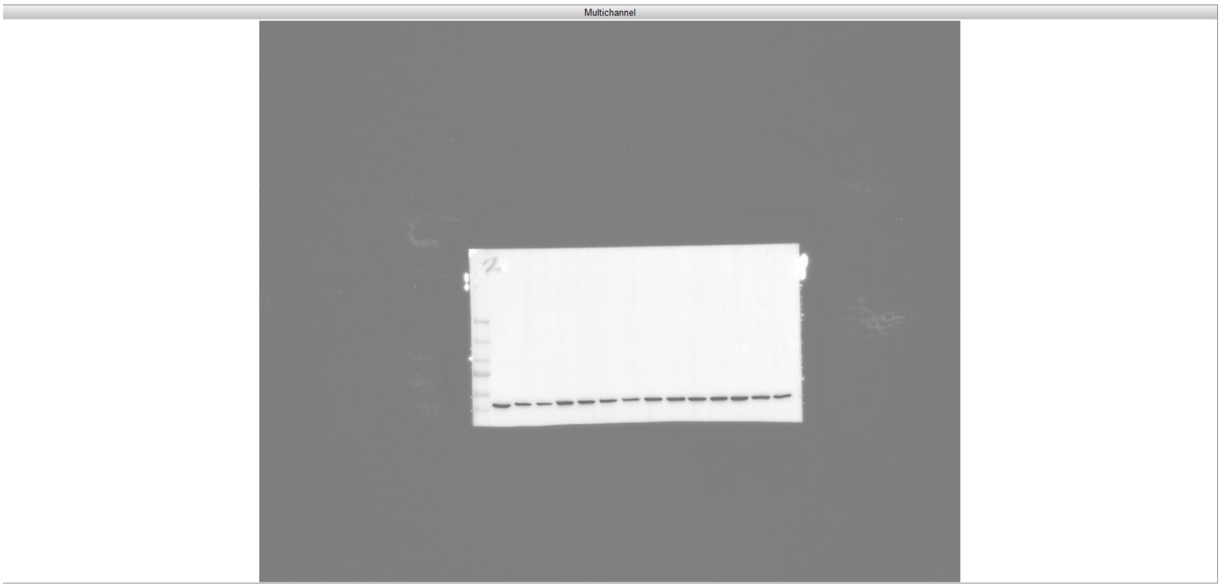

Image S16. Figure 4F. B-actin
